# Single-cell analysis of gene regulatory networks in the mammary glands of P4HA1-knockout mice

**DOI:** 10.1371/journal.pgen.1011505

**Published:** 2025-07-22

**Authors:** Akshat Gupta, Lilin Huang, Jinpeng Liu, Ke Chen, Ren Xu, Wei Wu

**Affiliations:** 1 Ray and Stephanie Lane Computational Biology Department, School of Computer Science, Carnegie Mellon University, Pittsburgh, Pennsylvania, United States of America; 2 Markey Cancer Center, University of Kentucky, Lexington, Kentucky, United States of America; 3 Department of Pharmacology and Nutritional Science, University of Kentucky, Lexington, Kentucky, United States of America; Tulane University School of Medicine, UNITED STATES OF AMERICA

## Abstract

Prolyl hydroxylation, catalyzed by collagen prolyl 4-hydroxylase (P4H), is a crucial post-translational modification involved in collagen biosynthesis. P4HA1, an isoform of P4H, plays a prominent role in stabilizing hypoxia-inducible factor-1α (HIF-1α). P4HA1 is frequently upregulated in highly aggressive triple-negative breast cancer, and has been implicated in tumor progression, metastasis, and chemoresistance. In this study, we investigated the role of P4HA1 in mouse mammary glands by analyzing gene regulatory networks (GRNs) in basal epithelial cells across two mouse groups: control (5Ht) and P4HA1-knockout (6Ho) mice. Specifically, we employed a single-cell network inference approach, integrating single-cell RNA sequencing with the SCENIC pipeline, and incorporated multiple validation strategies to construct gene regulatory networks (GRNs) specific to basal epithelial cells from each mouse group. Despite the inherent challenges of single-cell data, our approach identified reliable and reproducible GRN patterns across both the mouse groups. Based on these patterns, we identified subclusters of basal epithelial cells with similar regulatory profiles across the two mouse groups, as well as a unique subcluster in the control mice with a distinct regulatory pattern absent in the P4HA1-deficient 6Ho mice. This unique subcluster exhibited concurrent activation of stem cell development and inflammatory response pathways, suggesting the role of P4HA1 in regulating these biological processes linked to cancer initiation and progression. We verified these findings through multiple approaches, including in silico validation using multiple external datasets as well as experimental validation. Given that the loss of P4HA1 may disrupt stem cell development and inflammation response, our results suggest that targeting P4HA1 may offer a promising therapeutic strategy for breast cancer treatment.

## Author summary

In this study, we investigated the role of P4HA1 in regulating transcriptional programs of mammary basal epithelial cells in mice using a single-cell network approach. P4HA1 is a key enzyme involved in collagen production, which is essential for maintaining the structure of normal tissues. However, it also helps stabilize the hypoxia-inducible factor-1α (HIF-1α) protein that responds to low oxygen levels, a condition often found in various cancers. We generated and analyzed single-cell RNA sequencing data to understand the regulatory roles of P4HA1 in two groups of mice: one with a single functional allele of P4HA1 and the other with the P4HA1 gene knocked out. We constructed gene networks for basal epithelial cells in both groups using the SCENIC pipeline. Due to the inherent complexity and noise within single-cell data, we employed multiple validation strategies to ensure that the identified regulatory patterns and results in the networks across both mouse groups were reliable and reproducible. Our results suggest that the P4HA1 gene plays an important role in pathways related to stem cell development and inflammation, which are essential for both tissue morphogenesis and repair, as well as cancer progression. One unique subcluster of basal epithelial cells identified only in control mice with P4HA1 showed simultaneous activation of stem cell and pro-inflammatory processes, suggesting that this cell population plays a significant role in regulating these important cellular processes. Our work provides new insights into how P4HA1 might serve as a key regulator of mammary gland development and a potential target to slow down or prevent the progression of aggressive cancers.

## Introduction

Prolyl hydroxylation is a critical post-translational modification that enhances protein folding and stability, primarily by influencing collagen biosynthesis [1]. This modification is catalyzed by the enzyme collagen prolyl 4-hydroxylase (P4H). We and others have shown that P4HA1, an isoform of P4H, enhances the stability of hypoxia-inducible factor-1α (HIF-1α), a vital regulator of the cellular response to hypoxia, which promotes angiogenesis, cancer metastasis, and chemoresistance [2,3]. P4HA1 is frequently upregulated in cancer, particularly in triple-negative breast cancer (TNBC), a subtype associated with poor prognosis and aggressive growth [4,5].

It has been reported that basal epithelial cells play a crucial role in the normal mammary gland, and that their interactions with the tissue microenvironment can influence mammary gland morphogenesis [6,7]. In this study, we aimed to investigate the role of P4HA1 in basal epithelial cells of mouse mammary glands (MMGs) in two distinct mouse groups. The first group, referred to as 5Ht, has one functional allele of P4HA1 and serves as the control, while the second group, referred to as 6Ho, has a P4HA1-knockout where P4HA1 expression is silenced in basal mammary epithelial cells.

The advent and widespread adoption of single-cell RNA sequencing (scRNA-seq) datasets have spurred the development of various inference methods for reconstructing gene regulatory networks (GRNs) [8]. GRNs serve as graphical representations of biological systems, where nodes represent genes, and edges denote regulatory interactions between these genes [9], providing insights into complex regulatory mechanisms underlying cellular behaviors. However, the generation and inference of GRNs with single-cell transcriptomic data has long been challenging. As the volume of scRNA-seq data grows, effective and reliable network reconstruction becomes increasingly important. In particular, the SILGGM approach leverages Gaussian graphical models (GGMs), which estimate conditional dependencies between genes by transforming the problem of network estimation into a sparse estimation of precision matrices [10]. The information-incorporated Gaussian graphical model approach enhances traditional GGMs by integrating prior knowledge from existing studies, using penalization to balance observed data with supplementary information. This approach adaptively includes reliable gene connections while down-weighting potentially inaccurate information, improving inference accuracy and robustness [11]. Information-theoretic approaches, on the other hand, use measures such as mutual information to capture both linear and non-linear relationships between genes [12]. By considering higher-order interactions and multivariate dependencies, methods such as PIDC enable the inference of more complex regulatory networks by identifying synergistic and redundant information across groups of genes [12,13]. The Boolean networks method models genes as binary variables, and their interactions are modeled using logical operators; this method captures the dynamic behavior of GRNs by simulating state transitions through asynchronous updates [14].

In several benchmark studies [8,15], SCENIC [16] has emerged as a robust and scalable approach for gene network inference with single-cell data. Specifically, SCENIC first utilizes non-linear, non-parametric models such as Gradient Boosting regression to infer co-expression-based networks. It then uses motif enrichment analysis to identify transcription factors (TFs) and their direct target genes, refining the co-expression networks into regulons (a collective term referring to TFs and their target genes). Lastly, the activities of these regulons in individual cells are measured using an Area Under the Curve (AUC) metric. This metric ranks all genes in a cell based on their expression levels and examines if the genes in a regulon are near the top of that ranking. The more highly ranked the genes in the regulon are, the more active the regulon is considered in that cell.

In this study, we intended to investigate the transcriptional mechanisms underlying basal epithelial cells in the mammary glands of the control 5Ht and P4HA1-knockout 6Ho mice. Recent studies identified several subclusters of basal epithelial cells with potentially different underlying molecular mechanisms in the mouse mammary glands based on scRNA-seq data using cluster analysis [17,18]. A natural downstream task, therefore, is to compare the corresponding subclusters in the control and knockout mice to elucidate their transcriptional differences. However, to accomplish this, three main challenges need to be addressed. First, subclusters identified by cluster analysis based on scRNA-seq data can be affected by various factors [19]; e.g., depending on the number of cells, cell types, and cell ratios in the data, a clustering method can generate varying results. Such variations pose a significant challenge for downstream analyses, particularly when comparing subclusters between two groups of biological samples (e.g., 5Ht vs. 6Ho mice in this study), as it becomes difficult to distinguish whether the observed differences arise from technical biases introduced by the clustering method or reflect true biological variations between the groups. Second, subclusters identified based on gene expression profiles may contain subsets of cells with distinct underlying regulatory mechanisms, which can be particularly difficult to detect when these cell populations are small. Third, when comparing different groups of mice, aligning subclusters can be challenging, as differences in cell populations may lead to variations in underlying transcriptional programs and cellular compositions between individual mice.

To address these challenges, in this study, we employed an alternative GRN-based approach to identify subclusters of mammary basal epithelial cells in the 5Ht and 6Ho mice. We also developed a strategy that enabled us to align subclusters in the two mouse groups. Moreover, we utilized various validation strategies, including both in silico and experimental validation, to enhance the reliability of our findings from the GRN-based subcluster comparison in the two mouse groups. By doing so, we provide novel insights into the role of P4HA1 in regulating inflammation and immune responses, as well as stem cell differentiation within basal epithelial cells of the mouse mammary gland. Our findings reveal a distinct subcluster with both pro-inflammatory response and stem cell signatures in the basal epithelial cells of the control mice, which may potentially drive breast cancer initiation and development [20–22]. The absence of this subcluster in the P4HA1-deficient 6Ho mice suggests that removing P4HA1 disrupts these pathways in the knockout mice. Given the roles of inflammation in cancer development [22], our results indicate that inhibiting P4HA1 could offer a promising therapeutic strategy for treating breast cancer. Furthermore, our work suggests that the GRN-based subcluster identification and comparison provide an alternative approach to compare transcriptional mechanisms underlying diverse cell populations in different groups of biological samples.

## Results

**Fig 1:**
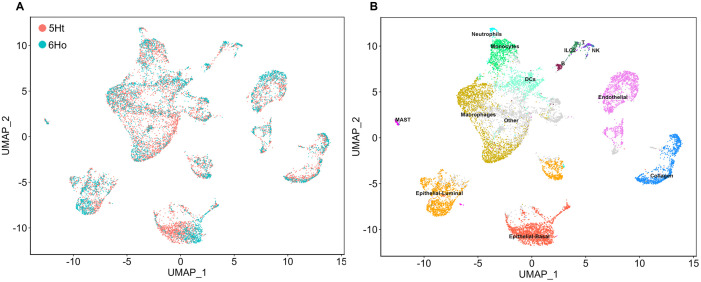
Integrated UMAP plots annotated by the 5Ht and 6Ho mouse groups (A) and by cell types (B).

In this study, we generated scRNA-seq data from the mammary glands of two groups of mice: the control 5Ht mice and the P4HA1-knockout 6Ho mice. Immunofluorescence images in S1 Fig show that P4HA1 expression in the 6Ho mice was silenced in K14-positive mammary epithelial cells. We integrated the single-cell data from the 5Ht and 6Ho mice using Seurat [23] to compare gene expression differences, shared cell states, and types between the two mouse groups. The Uniform Manifold Approximation and Projection (UMAP) plot [24] in Fig 1A shows that scRNA-seq data from both groups integrated well, overlapping nearly perfectly across different cell types. To visualize each mouse group individually while preserving the overall context of integration, we split the integrated UMAP plot by the mouse group (S2 Fig). We observed that the cell clusters in both the 5Ht and 6Ho mice remain highly similar in terms of shape and location.

Consistent with previous studies [18,25], the integrated UMAP segregates the three major cell types – epithelial basal and luminal cells, and macrophages – into distinct clusters (Fig 1B). The cell type-specific canonical markers for these clusters are shown in S3 Fig (also see Materials and Methods for details). The numbers of the cells in these three major cell types across both mouse groups are summarized in S1 Table. It can be seen that the number of basal epithelial cells and macrophages are substantially decreased in the 6Ho knockout mice, compared to the control mice (S1 Table).

### Network analysis

Next, we aimed to identify the transcriptomic differences underlying the mammary basal epithelial cells between the 5Ht and 6Ho mice through network and cluster analyses. We identified 289 and 290 regulons activated in the 5Ht and 6Ho basal epithelial cells using SCENIC, respectively. Of these regulons, 245 were regulated by the common TFs between the two mouse groups (shown in S2 Table). The remaining regulons in the basal epithelial cells of each mouse group were identified as unique.

### Cluster analysis

Since our goal was to understand the transcriptional regulatory differences between the two mouse groups, we sought to identify subclusters of the basal epithelial cells based on the regulon activities in these mice. In particular, we utilized the 245 common regulons to perform cluster analysis; since the TFs regulating these regulons were identified through network analysis in both the mouse groups, we considered these regulons more reliable than the unique regulons detected only in one mouse group.

Aligning basal epithelial subclusters in the 5Ht and 6Ho mice

**Fig 2:**
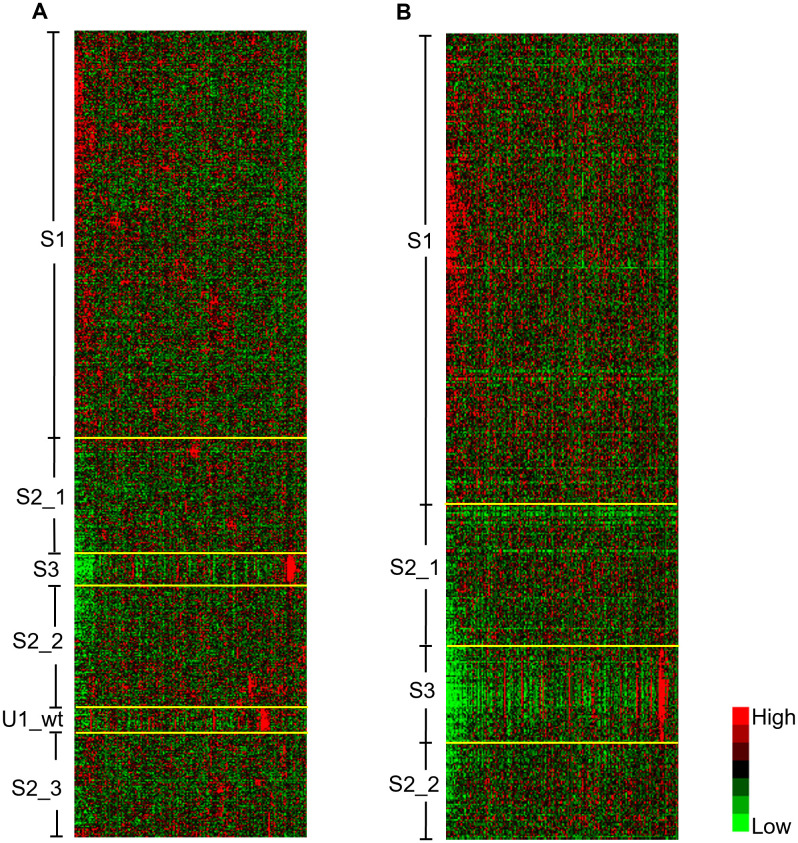
Subclusters of the basal epithelial cells in 5Ht and 6Ho mice revealed by hierarchical clustering based on activities of the common regulons. Heatmap showing the basal epithelial subclusters and the regulon patterns of (A) the 5Ht mice, and (B) the 6Ho mice. Cells are shown in rows; the 245 common regulons detected in both the mouse groups are shown in columns.

Our cluster analysis revealed 4 subclusters of basal epithelial cells in the control mice, and 3 subclusters in the knockout mice (Fig 2). To compare them, we first needed to align the subclusters. While most of the cells in both mouse groups should be of the same types, the differences between these groups may lead to distinct regulatory behaviors and gene expression patterns, making alignment challenging. Nevertheless, we reasoned that since basal epithelial cells in the 6Ho mice are P4HA1-deficient, some subclusters of cells should exhibit different regulatory patterns compared to their counterparts in the 5Ht mice, while others should remain similar. Thus, we employed the following strategy to align the basal epithelial subclusters in the two mouse groups.

We first clustered the basal epithelial cells of the 5Ht mice and their regulons, respectively, using hierarchical clustering (S4A Fig). Then, we clustered the basal epithelial cells of the 6Ho mice, also with hierarchical clustering. Subsequently, in order to visualize the patterns of the common regulons in the two mouse groups, we reordered the regulons in the 6Ho mice in the same order as the clustered regulons in the 5Ht mice, without any further clustering (S4B Fig, see Materials and Methods for details).

After reordering the basal epithelial cells and their regulons in the 5Ht and 6Ho mice, respectively, several subclusters in the two mouse groups displayed similar regulon activity patterns as shown in Figs 2 and S4, allowing them to be readily aligned. Specifically, hierarchical clustering of the basal epithelial cells yielded two large subclusters, S1 and S2, in the 5Ht and 6Ho mice, each of which displays a similar regulon activity pattern between the two mouse groups. It is also evident that there are two subclusters – which we name S3 and U1_wt – nested in subcluster S2 of the 5Ht cells, and one subcluster S3 nested in subcluster S2 of the 6Ho cells. Subcluster S3 showed similar regulon activity patterns in the 5Ht and 6Ho mice.

Other than the “similar” subclusters that are present in both mouse groups, it was also evident that one subcluster, U1_wt, of the 5Ht mice did not have any corresponding subcluster with a similar regulon pattern in the 6Ho mice, suggesting that depletion of P4HA1 from basal epithelial cells had a direct impact on this subcluster of the cells in the knockout mice, which may lead to the loss or significant alteration of certain regulon activities in these 6Ho cells. Overall, using this subcluster alignment strategy, we identified subclusters S1–S3 and their counterparts in the basal epithelial cells of the 5Ht and 6Ho mice. The number of cells in each subcluster of the two mouse groups is summarized in S3A Table.

Notably, hierarchical clustering is not typically considered ideal for partitioning single cells, as it can sometimes produce fragmented clusters with scRNA-seq data [26]. Nevertheless, we found this approach to be particularly useful for subcluster alignment, as its hierarchical structure allowed us to uncover patterns within the data and detect subtle signals, even from small subclusters of cells.

To ensure that subclusters S1–S3 in both mouse groups, as well as the 5Ht-specific U1_wt subcluster, represent bona fide basal epithelial cells without contamination from other cell types, we examined the expression of canonical basal cell markers within these clusters (see Materials and Methods for details). As shown in S5 Fig, epithelial basal markers are strongly expressed across all subclusters of both mouse groups, while markers specific to immune cells (Ptprc) and macrophages (Adgre1) are absent. These expression patterns confirm that the subclusters are exclusively composed of basal epithelial cells and that their regulon activities are not confounded by contamination from immune cells, including macrophages.

Furthermore, we employed a more quantitative approach to verify the correctness of the subcluster alignment. First, we examined the identified subclusters based on their AUC scores with the UMAP plots. As shown in S6 Fig, subclusters S1 and S2 align very well with their counterparts in the two mouse groups. Interestingly, subclusters S3 (from both 5Ht and 6Ho mice) and U1_wt (from 5Ht) appeared as two outlier subclusters in S6 Fig. To measure the similarity between these subclusters quantitatively, we calculated the pairwise distances between the centroids of the 5Ht and 6Ho subclusters (see Materials and Methods for details). As shown in S4 Table, the centroids of subclusters S1–S3 indeed have the shortest distance with their counterpart in the respective mice; on the other hand, the centroid of subcluster U1_wt of the 5Ht mice was not close to that of any 6Ho subcluster. These results supported the validity of the aligned subclusters in the two mouse groups.

For further comparison, we also examined the distributions of basal epithelial subclusters in the 5Ht and 6Ho mice using UMAP plots generated from scRNA-seq expression profiles. As shown in S7 Fig, with the exception of subcluster S3 whose cells from both mouse groups mapped to a similar region in the UMAP space, the other subclusters (S1, S2 and U1_wt) did not form distinct clusters in the UMAP plots—despite exhibiting distinct regulon activity patterns as shown in Fig 2. Collectively, these results suggest that the subclusters identified based on regulon activity patterns differ from those derived from the scRNA-seq expression profiles, thereby offering an alternative perspective for understanding transcriptional regulation of the cells.

### Validation of our results

To ensure that the network results we generated for the 5Ht and 6Ho mice were accurate and reliable, we validated the TF-driven regulons detected in these mice using the Mouse Genome Informatics (MGI) database [27].

We first curated a set of 1032 TFs active in mouse mammary glands using the MGI database. Then, we compared the regulons identified in the basal epithelial cells of the 5Ht and 6Ho mice to this set of TFs. The results are summarized in S2 Table. Of the 289 TF-driven regulons detected in the 5Ht basal epithelial cells, 242 TFs (~84%) overlapped with the MGI set. Similarly, of the 290 TF-driven regulons detected in the 6Ho basal epithelial cells, 244 TFs (~84%) overlapped with the MGI set. We also examined the TFs regulating each mouse group’s unique and common regulons to see how they overlap with the MGI set. A high overlap with the MGI set was observed for these TFs in both mouse groups (S2 Table).

On the other hand, among the 196 TFs included in the network analysis but not detected as regulons in the 5Ht cells, only 55 (~28%) were part of the MGI set. Similarly, of the 195 TFs not detected as regulons in the 6Ho cells, only 53 (~27%) were in the MGI set. These findings indicate that the TF-driven regulons identified using SCENIC are biologically valid, aligning with TFs known to be active in mouse mammary gland cells as curated in the MGI database.

### Comparing basal epithelial subclusters in the 5Ht and 6Ho mice

First, we compared the basal subclusters of the two mouse groups using differential gene expression (DGE) analysis. For each “similar” subcluster, differentially expressed genes (DEGs) were identified between the 5Ht and 6Ho basal epithelial cells of that subcluster. In contrast, for the “unique” subcluster U1_wt in 5Ht mice, DEGs were identified between the basal epithelial cells of this subcluster and the remaining basal epithelial cells of the same mouse group.

“Similar” subclusters S1-S3 between 5Ht and 6Ho mice: Amongst all the “similar” subclusters, we found that subcluster S1 had the lowest number of DEGs, while subcluster S3 had the highest (S3B Table), suggesting that S1 is most similar between the two mouse groups. Our functional enrichment analysis showed that many genes involved in regulation of protein stability, response to unfolded protein and chaperone-mediated protein folding were significantly enriched among upregulated genes in 6Ho mice in each of the “similar” subclusters (S5A–S5C Table). It is known that P4HA1-mediated hydroxylation plays an essential role in proper collagen folding in the cell [2], our enrichment results, therefore, are consistent with the fact that depletion of P4HA1 in the 6Ho mice causes collagen to be misfolded and degraded due to lack of hydroxyproline. Moreover, genes involved in cellular respiration, oxidative phosphorylation, glycolysis, and various mitochondrial activities (e.g., mitochondrial respiratory chain complex assembly) were also significantly enriched among upregulated genes in each of the “similar” subclusters of the 6Ho mice (S5A–S5C Table). For instance, in subcluster S1, our results showed that 38 genes involved in cellular respiration, 30 genes in oxidative phosphorylation, and 8 genes in glycolysis were upregulated in the 6Ho mice compared to the control mice (S5A Table). We observed similar results in subclusters S2 and S3, suggesting that these cellular processes are prevalent across the majority of basal epithelial cells in both mouse groups. Our results also revealed a significant enrichment of the genes involved in endoplasmic reticulum stress response among DEGs in subcluster S2 (S5B Table), and an enrichment of the genes participating in oxidative stress among DEGs in subcluster S3 (S5C Table).

An examination of the Hif1a expression level in these data revealed that Hif1a is downregulated in subcluster S3 of the 6Ho mice with a fold change of 0.65 and a nominal p-value of 0.02. Together, these results agree with our previous study showing that P4HA1 regulates ROS and oxidative phosphorylation via the HIF-1 pathway in breast cancer cells [5]. In particular, upregulation of the P4HA1 in breast cancer cells increased HIF-1 protein stability, accompanied by decreased respiration, glycolysis, oxidative phosphorylation, and reactive oxygen species (ROS) levels in breast cancer cells [5].

**Fig 3:**
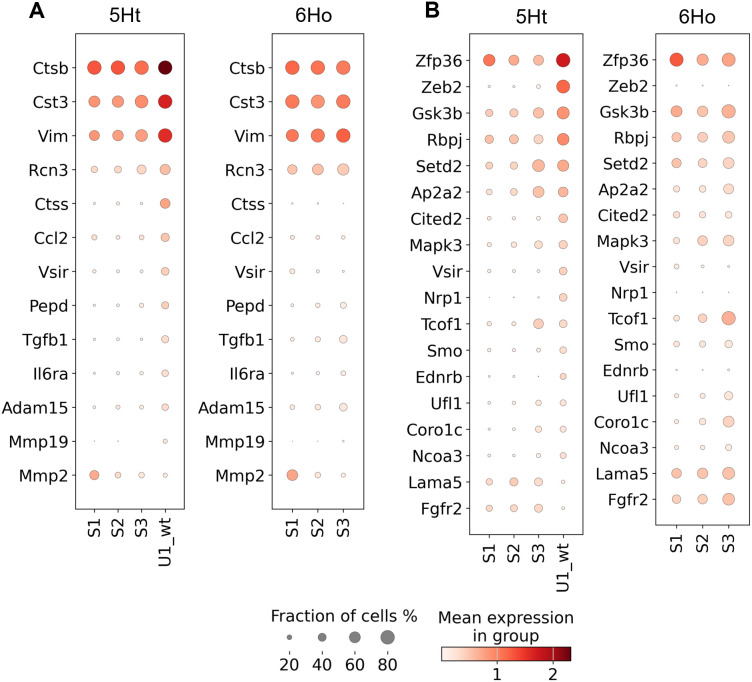
DEGs between subcluster U1_wt and the other 5Ht basal epithelial cells enriched in collagen and stem cell pathways. Dot plots display the DEGs enriched in (A) collagen metabolic, and (B) stem cell differentiation pathways. DEGs are sorted in descending order based on the fraction of the cells in subcluster U1_wt in which they are expressed.

Unique subcluster U1_wt in the control mice: Our GO analysis showed that 13 genes involved in the collagen metabolic process were significantly enriched among upregulated genes DEGs in these cells (S6A Table, Fig 3A). This result is consistent with the fact that collagen cannot be properly synthesized in 6Ho mice due to a lack of P4HA1, which explains why this subcluster only exists in 5Ht mice.

Moreover, we found that 18 DEGs, including Zeb2 (one of the top 10 most significant DEGs), were involved in stem cell differentiation and significantly enriched among DEGs in this subcluster (S6A Table, Fig 3B). Of these 18 DEGs, 16 were found to be upregulated in this subcluster. This result agreed with our previous findings showing that upregulation of P4HA1 in breast cancer cells increased HIF-1 protein stability, subsequently enhancing the stemness of the cells [5]. Additionally, our analysis also showed that 21 genes involved in the regulation of cell morphogenesis and 34 genes associated with angiogenesis were significantly enriched among the DEGs detected in this subcluster (S6A Table).

Unlike similar clusters, however, our results also show that subcluster U1_wt was significantly enriched with many upregulated genes involved in numerous inflammatory and immune response processes, e.g., acute inflammatory response, positive regulation of macrophage migration and chemotaxis, macrophage activation, B cell, and T cell-mediated immunity (S6B Table).

Notably, after we identified this basal epithelial subcluster enriched with inflammatory genes in the 5Ht mice, we found that a recently published mouse aging atlas study reported a similar pro-inflammatory myoepithelial subcluster (Epi-C11) [28]. In particular, of the 8 pro-inflammatory and interferon-gamma signature genes expressed in subcluster Epi-C11, highlighted in that study, four – Cd74, H2-Ab1, H2-Eb1, and Ccl2 – were also upregulated in the 5Ht U1_wt subcluster (S8 Fig). The upregulation of these overlapping inflammatory genes, together with the shared transcriptional circuitry between the pro-inflammatory myoepithelial subcluster and the U1_wt (described below), provides cross-study confirmation that the U1_wt cells represent a pro-inflammatory state within the basal epithelial cell population of the 5Ht mice.

Significant regulons and differentially expressed target genes (DETGs)

To further identify differences between the 5Ht and 6Ho mice, we quantified the activity of each regulon within individual cells of these mice using the AUC scores with SCENIC. This score assesses how strongly a TF-driven regulon is active in each cell. We then focused our analysis on DETGs, which are target genes of the TF within a regulon that are differentially expressed between the corresponding subclusters of the 5Ht and 6Ho mice. We reasoned that these genes drive distinct regulatory activities of each regulon in the two mouse groups, thus shedding light on how these biological processes are regulated in the cells of these subclusters.

Our results show that subclusters S1 and S2 had the lowest numbers of significantly different regulons amongst all the subclusters between the two mouse groups, indicating that similar transcriptional mechanisms drive the corresponding subclusters in the control and the knockout mice. In contrast, subclusters S3 and U1_wt had the highest numbers of significantly different regulons (S3B Table). Our results show that the activity of the Hif1a regulon in the S1 cells of the 6Ho mice is significantly lower than that in 5Ht mice (adjusted p value = 0.01), again consistent with our previous study showing that upregulation of P4HA1 in aggressive breast cancers leads to the increased level of HIF-1α in the mammary tissues of the patients [5]. The Hif1a regulon in the S3 cells also has a lower activity (but not statistically significant) in the 6Ho mice.

Since our goal is to elucidate transcriptional differences underlying mammary basal epithelial cells in the control and the P4HA1-knockout mice, we subsequently identified and characterized the regulons potentially playing roles in key signaling pathways in subcluster S3 of the two mouse groups and subcluster U1_wt of the 5Ht mice.

Subcluster S3 of the 5Ht and 6Ho mice: Our analyses revealed that the percentage of basal cells in subcluster S3 of the 6Ho mice is 1.5 times higher than that in the 5Ht mice (S3A Table). Moreover, several regulons in subcluster S3 regulate DETGs that were enriched in potentially important signaling pathways in both the mouse groups. For example, among the DETGs of a significantly different regulon E2f1, those participating in mitochondrion organization and carbohydrate derivative metabolic processes were significantly enriched and downregulated in the control mice (S7A Table). In contrast, the DETGs of this regulon involved in rRNA and peptide metabolic processes were significantly enriched and upregulated in the knockout mice (S7B Table). These findings implicate E2f1 as a key regulator of various metabolic processes in response to the loss of P4HA1 in this subcluster of the knockout mice.

**Fig 4:**
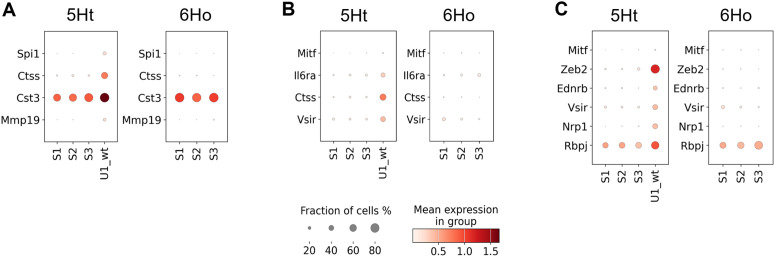
The regulons detected in subcluster U1_wt of the 5Ht mice with the DETGs involved in the collagen metabolic process and stem cell development pathway. Dot plots show paired comparisons of the (A) Spi1 and (B) Mitf regulons, with their DETGs participating in collagen metabolic pathways, and (C) Mitf regulon with its DETGs participating in the stem cell differentiation, across the basal epithelial subclusters of the 5Ht and 6Ho mice. DETGs are sorted by their ranks within their respective 5Ht regulons.

Subcluster U1_wt of the 5Ht mice: Genes involved in the collagen metabolic and catabolic processes were significantly enriched among the DETGs regulated by the two significantly different regulons Spi1 and Mitf in these cells (S8A Table; Fig 4A & 4B). Both these regulons were among the top significantly different regulons, with the Spi1 regulon being the most significantly different in this subcluster. Moreover, we identified significant enrichment of 5 DETGs regulated by Mitf – Zeb2, Ednrb, Nrp1, Rbpj, and Vsir – which were involved in the stem cell development pathway (S8B Table). It can be seen in Fig 4C that their expression levels are upregulated in subcluster U1_wt, compared to those in the other subclusters of the two mouse groups.

### Inflammatory and immune responses

**Fig 5:**
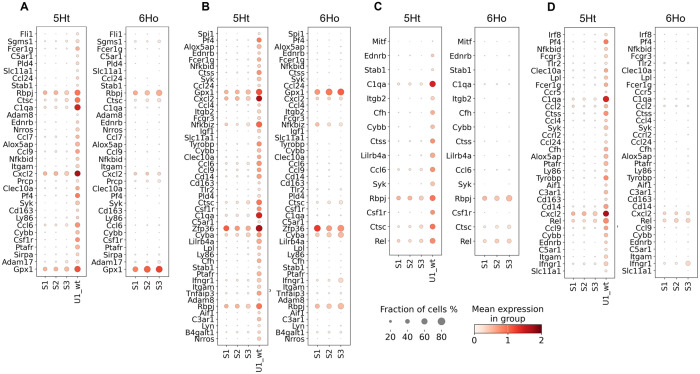
Dot plots illustrating four regulons in subcluster U1_wt of the 5Ht mice with the DETGs participating in the inflammatory response pathways. Dot plots show paired comparisons of the (A) Fli1, (B) Spi1, (C) Mitf, and (D) Irf8 regulons, and their DETGs across the basal epithelial subclusters of the 5Ht and 6Ho mice. Panels (A-D) show the DETGs involved in inflammatory response, which are sorted by their ranks within their respective 5Ht regulon.

Using SCENIC, we identified 15 significantly different regulons between the two mouse groups that showed a significant enrichment of the DETGs participating in inflammatory and immune-responses (S9 Table, Fig 5). Among these regulons, 9 regulons also regulate DETGs with significant enrichment of the genes involved in macrophage activation and migration (S9 Table).

**Fig 6:**
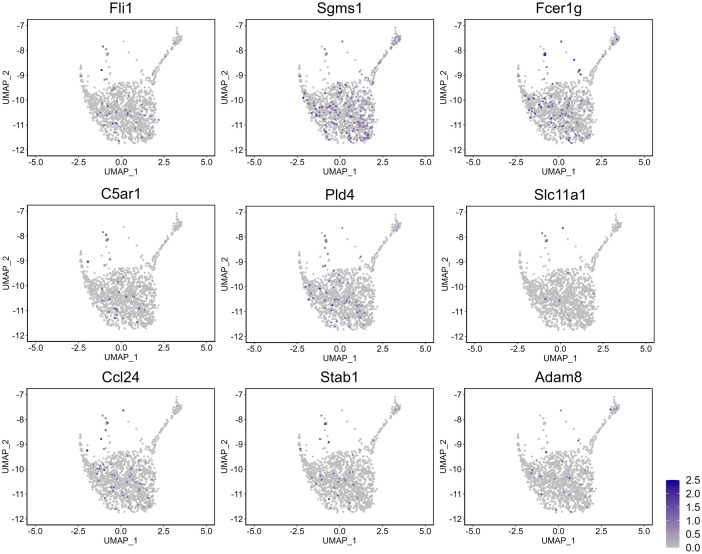
Feature plots of the Fli1 regulon and its DETGs participating in the inflammation response pathway in subcluster U1_wt of the 5Ht mice. Expression levels of Fli1 (TF) and several DETGs (within the regulon) detected in the cells of subcluster U1_wt are shown across all basal epithelial cells of the 5Ht and 6Ho mice.

Notably, of these 15 regulons, Fli1 regulated 32 DETGs involved in the inflammatory response pathway – all of which were upregulated in subcluster U1_wt (Fig 5A) – 9 of these DETGs were also found to participate in macrophage activation and chemotaxis. Feature plots in Fig 6 show the distributions of the 8 DETGs in the Fli1 regulon across all basal epithelial cells of the 5Ht and 6Ho mice; it can be seen that their expression levels align very well with the expression of Fli1 in the 5Ht U1_wt cells. A literature review highlighted Fli1 as a key proto-oncogene with crucial roles in hematopoiesis and vascular development [29]. Its overexpression has been implicated in highly malignant triple-negative breast cancer, where it promotes tumor progression by driving proliferation, inhibiting differentiation, and enhancing cellular survival under hypoxic conditions [30–32].

We also found that, among the TFs driving the 15 significantly different regulons with the pro-inflammatory DETGs, Spi1 and Mitf (Fig 5) each regulated DETGs involved in the collagen metabolic processes (S8A Table, Fig 4A & 4B). Within the same subcluster, we also found significant enrichment of DETGs regulated by Mitf participating in the stem cell development pathway (S8B Table, Fig 4C).

Taken together, these results show that multiple TFs regulate pro-inflammatory DETGs in subcluster U1_wt of the control mice, and that TFs such as Spi1 and Mitf may mediate potential crosstalks between inflammatory responses, macrophage activation and migration, as well as collagen metabolism, and stem cell development in this subcluster of the 5Ht cells. Furthermore, the presence of this subcluster with pro-inflammatory and stem cell signatures in the control mice, while absent in the knockout mice, supports the notion that expression of P4HA1 in the control mice leads to increased stemness and inflammation in this subcluster of the cells.

### External and experimental validation

External validationFollowing our findings above, a recent mouse aging atlas study reported a myoepithelial subcluster Epi-C11 with similar expression of pro-inflammatory genes in mouse mammary glands [28]. Fli1 was highlighted in this study and was found to be expressed in MMGs with accessible chromatin in both 3-month-old (3M) and 18-month-old (18M) mice by single-nucleus assay for transposase-accessible chromatin with sequencing (snATAC-seq). The differentially accessible (DA) peak between the two mouse groups detected in the promoter region of Fli1 also contained binding motifs for Irf4 and Irf8, which agrees with our finding showing the activation of the Fli1, Irf4, and Irf8 regulons in the U1_wt subcluster of the control mice. Additionally, among the 15 pro-inflammatory regulons we identified (S9 Table), 6 of them (Fli1, Irf8, Rel, Stat1, Jund, Runx3) showed peak opening in the promoter region while 6 (Spi1, Mitf, Klf2, Maf, Irf5, Pparg) showed openings in other regions (such as exons and introns) in the mouse aging atlas study (S10A Table).

We also verified other results using the data provided by the mouse aging atlas study. In particular, among 81 significantly different regulons we detected, 72 TFs showed peak accessibility in various genomic regions—54 TFs exhibited peaks in promoter regions and 18 in other regions (e.g., exons and introns) (S10A Table). Additionally, 42 of these TFs were expressed in the Epi‑C11 cluster of 3‑month‑old mice—matching the age of the mice used in our study (S10B Table).

Moreover, among 534 DEGs in U1_wt, i) 370 genes have opening peaks in various regions, with 298 genes having peak openings in the promoter region and 72 additional genes in other regions (S11A Table); ii) 311 genes were expressed in 3M mice in Epi-C11 (S11B Table); and iii) 64 genes, including Cd74, H2-Ab1, H2-Eb1, H2-Aa, Ccl2, and Ccl7, were also differentially expressed between Epi-C11 and other myoepithelial cells in the mouse aging atlas study (S11C Table). Taken together, these results indicate that subcluster U1_wt shares substantial similarity with subcluster Epi-C11 in the mouse aging atlas study, thus supporting the validity of our findings.

To further investigate whether significantly different regulons and DEGs in U1_wt are linked to breast cancer, we examined two external publicly available microarray datasets, GSE65194 [33] and GSE45827 [34], from the Gene Expression Omnibus (GEO) database. These datasets were generated from the same cohort of 41 triple-negative breast cancer samples and 11 normal samples from healthy breast tissues [33,34]. We found that of the 81 TFs that regulate significantly different regulons, 51 were differentially expressed between the TNBC and normal samples (S12 A, & S12B Table), including 9 pro-inflammatory TFs (Mitf, Irf5, Irf7, Stat1, Rel, Klf2, Jund, Runx3, and Maf) shown in S9 Table. Also, of the 534 DEGs in U1_wt, 315 were differentially expressed between the TNBC and normal samples (S12C & S12D Table), including Cd74 and Ccl2. These findings indicate that the transcriptional modules active in subcluster U1_wt shares notable similarities with gene expression patterns observed in the human TNBC samples, suggesting that the U1_wt regulons may play a functional role in tumor initiation, progression, and immune-related processes associated with TNBC.

Experimental validationSince our results showed that several regulons in the 5Ht U1_wt subcluster upregulated DETGs enriched in inflammatory response and macrophage migration (S9 Table), and given the crucial role of macrophages in the inflammatory response and their potential involvement in promoting breast cancer initiation within premalignant mammary lesions [35], we performed immunohistochemistry (IHC) to examine macrophage accumulation in the mammary glands of the 5Ht and 6Ho mice.

**Fig 7:**
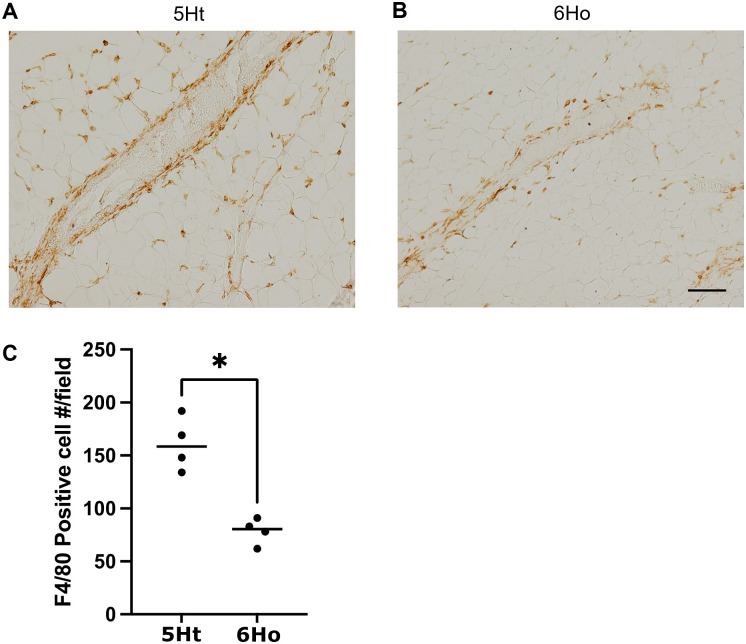
Immunostaining of macrophages in the mammary glands of the 5Ht and 6Ho mice. Macrophages were identified by the F4/80 antibody, shown by brown staining. Representative figures show the presence of macrophages in the mammary tissue of (A) 5Ht mouse, and (B) 6Ho mouse. Scale bar, 100 µm. (C) Quantification of F4/80-positive macrophages in the IHC staining images; n = 4 mice per group, *p < 0.05.

To identify macrophages, we immunostained sections of the mouse mammary gland with F4/80 antibodies [36]. Representative images in Fig 7 showed that the mammary tissue of the 5Ht mouse has a higher presence of macrophages (Fig 7A), which may indicate more active immune involvement. On the other hand, the mammary tissue of the 6Ho mouse has fewer macrophages (Fig 7B), suggesting that the P4HA1 deficiency could be linked to a reduction in macrophage-driven inflammation in these mice. This observation was confirmed by quantification of the F4/80 positive cells in immunohistochemistry (IHC) staining from 4 pairs of mice (Fig 7C), and is also consistent with our scRNA-seq analysis revealing a substantial decrease in the number of macrophages in the mammary glands of the 6Ho mice compared to those of the 5Ht mice (S1 Table). Together, these findings validated our results, confirming that macrophage activation is indeed disrupted in the P4HA1-deficient mice, thus reinforcing the critical role of P4HA1 in supporting macrophage-mediated inflammatory processes in mouse mammary tissues.

## Discussion

In this study, we investigated the transcriptional mechanisms of P4HA1 in mammary basal epithelial cells of the control 5Ht and P4HA1-knockout 6Ho mice using scRNA-seq data. Using a GRN-based clustering approach, followed by subcluster alignment, we identified three “similar” subclusters of basal epithelial cells in the two mouse groups, as well as one unique subcluster, U1_wt, in the control mice. Our GRN analyses revealed distinct regulatory differences between the basal epithelial subclusters of the two mouse groups. Specifically, in the subclusters (S1-S3) common to both mouse groups, the loss of P4HA1 in the 6Ho mice altered several important pathways including protein folding, respiration, oxidative phosphorylation, glycolysis, and mitochondrial activities, as well as significantly reduced Hif1a regulon activities in the S1 cells and downregulated Hif1a expression in the S3 cells. These results agree with our previous study, which showed that upregulation of the P4HA/HIF-1 axis enhances cancer cell stemness, accompanied by decreased oxidative phosphorylation and ROS levels [5]. In contrast, our identification of the unique subcluster U1_wt specific to the control mice – enriched with DEGs and DETGs in stem-cell development and pro-inflammatory responses – suggests a distinct basal epithelial cell state that is dependent on P4HA1.

Our GRN-based clustering approach enabled us to identify subclusters governed by the shared regulatory mechanisms, ensuring that the observed patterns reflected genuine biological processes rather than artifacts introduced by noise or technical variability. As our results have shown, this approach can be particularly useful for detecting a small number of cells with a distinct regulatory mechanism, which could otherwise be missed by the conventional clustering methods based on scRNA-seq expression profiles. For example, our approach identified subclusters S3 and U1_wt, which are clearly distinct – albeit small – subclusters in the UMAP plot based on regulon activities and are worth further investigation. These subclusters may not be able to be detected by the conventional clustering methods for cell type identification. Our approach, thus, offers an alternative strategy for identifying functionally relevant cell subclusters.

One of the significant challenges in single-cell network analysis is the inherent noise and technical variability associated with the scRNA-seq data. This problem is compounded when comparing networks from different biological conditions, such as the 5Ht and 6Ho mouse groups, where distinguishing genuine biological differences from technical artifacts becomes critical. To address these challenges, we employed several validation strategies throughout this work. First, we carefully aligned subclusters of the basal epithelial cells in both 5Ht and 6Ho mice using reproducible regulon activity patterns (Fig 2), and confirmed their similarity using the UMAP plots based on the regulon activities of the subclusters (S6 Fig) as well as the centroid analysis (S4 Table). Second, we validated the regulon TFs against curated public databases, such as the MGI database, focusing on TFs known to be active in the mouse mammary gland. We did so to ensure that the detected TFs and their regulons were biologically valid. Third, we cross-validated our results in the control mice with our previously published study [5] and a recently published mouse aging atlas study [28], ensuring that our findings aligned with known biological knowledge. Finally, we verified our results using two external human datasets comprising TNBC and healthy breast tissue samples, confirming that the regulatory modules uncovered in our mouse model translate to human disease contexts.

Furthermore, we performed the experimental validation using immunohistochemistry to assess the macrophage accumulation in the mammary glands of the two mouse groups. We observed a significant reduction in macrophage presence in the mammary tissues of the 6Ho mice, compared to those of the 5Ht mice (Fig 7). This result is consistent with our scRNA-seq data which showed a substantial decrease in the number of macrophages in the 6Ho mice (S1 Table). These findings support the notion that the absence of P4HA1 suppresses macrophage activation and migration in the knockout mice.

Together, these validation strategies ensured that our findings with the reconstructed networks were robust and reflective of genuine biological differences between the 5Ht and 6Ho groups, mitigating concerns about noise and technical artifacts resulting from the scRNA-seq data.

Notably, our results revealed a unique subcluster (U1_wt) of the basal epithelial cells in the control mice with upregulated pro-inflammatory signature genes, as well as genes involved in macrophage activation and migration. This subcluster resembles a pro-inflammatory myoepithelial subcluster reported in a recent mouse aging atlas study. Moreover, our study also identified redundant functions of multiple TFs in regulating inflammatory responses and macrophage migration. We found that 15 TFs, including Fli1, Spi1, Mitf, and Irf8, regulated overlapping DETGs involved in these processes, creating a highly redundant regulatory network. A literature review further emphasized critical functions of Fli1 as a proto-oncogene, implicating it as one of the ten key regulators of blood stem and progenitor cells [29,37]. The dysregulation of Fli1, observed in aggressive TNBC cell lines [32] suggests its potential role in driving breast cancer progression. Additionally, it has been known that Spi1 encodes a master TF that plays crucial roles in hematopoiesis and cell fate decisions [38], while Mitf encodes microphthalmia-associated transcription factor that plays a key role in immune responses [39]. Interestingly, Spi1 and Mitf function synergistically during osteoclast differentiation [40,41], consistent with our results showing both of them regulating DETGs involved in immune responses in the 5Ht U1_wt cells. Moreover, many studies have shown that functional redundancy is critical for maintaining cellular stability, particularly in dynamic environments such as tumorigenesis [42,43]. It provides compensatory regulation when the functions of individual TFs are disrupted, ensuring that critical processes, such as immune responses and stem cell differentiation, continue unhindered [44]. Additionally, the Spi1 and Mitf regulons also contain an enrichment of the DETGs involved in collagen metabolic process and stem cell development, suggesting potential crosstalk between these processes in the U1_wt cells. Although the pro-inflammatory U1_wt subcluster was found in the control mice in this study, substantial evidence from various cancer studies indicates that chronic inflammation can drive tumorigenesis and growth [45]. Our investigation using two external human microarray datasets showed that a considerable number of significantly different regulon TFs and DEGs in U1_wt were also differentially expressed between the TNBC and normal samples, suggesting that the transcription network and the DEGs in this subcluster play potentially significant roles in driving TNBC initiation and development.

While our study provides novel insights into the molecular mechanisms of P4HA1, it is important to acknowledge several limitations. In this work, we used the mice with a single-allele P4HA1 deletion (5Ht) as our control group because we did not observe significant differences in mammary gland morphogenesis between wild-type and 5Ht mice. Nevertheless, we cannot entirely rule out the possibility that the loss of a single P4HA1 allele may affect transcriptional regulation in the basal epithelial cells of the mouse mammary gland. Additionally, we controlled for age by analyzing three pairs of 8‑week‑old 5Ht-6Ho mice from the same litters to minimize variation; however, we did not account for the estrous cycle, which may influence epithelial cell states. Future studies incorporating estrous cycle staging would provide a better understanding of the transcriptional changes associated with P4HA1 deficiency.

Moreover, the identified pro-inflammatory U1_wt basal subcluster in control mice may raise concerns of potential macrophage contamination within the basal epithelial cell population. To mitigate such concerns, during the pre-processing step for our network analysis, we removed all the basal epithelial cells expressing canonical marker genes for immune cells (Ptprc) and macrophages (Adgre1). We also employed a reference-based cell type annotation method to cross-validate our clustering-based annotation results and remove all the basal cells potentially contaminated by other cell types, e.g., immune cells, fibroblasts, etc.

Furthermore, despite our extensive in silico validation of our results using publicly available databases and multiple external datasets, functional experiments – such as lineage tracing or perturbation of specific TFs within the identified subclusters – are still needed to validate the biological roles of these TFs in regulating the identified subclusters of the control and knockout mice in future studies. Additionally, while we did not extensively characterize mammary stem cells (MaSCs) or other immune cell populations in this work, these will also be areas of focus in our future investigations.

By leveraging single-cell network inference techniques and validating our findings through multiple strategies, we provide novel insights into the molecular mechanisms of P4HA1 in potentially regulating inflammation and immune response, as well as stem cell differentiation in the mouse mammary gland. The absence of the pro-inflammatory subcluster of basal epithelial cells in P4HA1-deficient 6Ho mice suggests that P4HA1 plays a key role in regulating pro-inflammatory responses in these cells and may serve as a potential therapeutic target for triple-negative breast cancer treatment.

## Materials and methods

### Ethics statement

The mouse experiments (Protocol Number: 2020–3639) were approved by the Institutional Animal Care and Use Committee (IACUC) at the University of Kentucky. All mouse experiments were performed with the Division of Laboratory Animal Resources guidelines at the University of Kentucky.

### Mammary gland isolation and dissociation for scRNA-seq data generation

In this study, we generated scRNA-seq data from two groups of mice: K14-cre; P4HA1 ^loxp/+^ (control, 5Ht) mice and K14-cre; P4HA1 ^loxp/loxp^ (P4HA1-knockout, 6Ho) mice. For each group, we used three pairs of 8-week-old mice, with each pair originating from the same breeding to minimize age and estrous cycle variations. P4HA1 expression was specifically silenced in the basal mammary epithelial cells in 6Ho mice.

To isolate mammary gland cells for scRNA-seq, we dissected mammary glands from both groups and mechanically dissociated them. The finely minced tissue was transferred to a digestion mix consisting of DMEM/F12 (Gibco) + 10 mM HEPES (Gibco) + collagenase (Roche) + 200 U ml ⁻ ¹ hyaluronidase (Sigma) + gentamicin (Gibco) for 3 hours at 37°C and vortexed every 30 minutes. After the lysis of red blood cells in NH_4_Cl, cells were briefly digested with warm 0.05% Trypsin-EDTA (Gibco), 5 mg ml^−1^ dispase (Sigma), and 1 mg ml^−1^ DNase (Sigma), and filtered through a cell strainer (BD Biosciences) to remove debris, yielding a single-cell preparation for sequencing.

Propidium iodide (PI, Sigma) was used at a working concentration of 1 μg/ml, prepared by diluting the stock solution 1:1000 to detect dead cells. After sorting, the cells were spun down and resuspended. Samples were manually counted using an improved Neubauer chamber, and the cell concentration was normalized. Equal numbers of cells per sample were processed for scRNA library preparation.

### ScRNA-seq data generation and initial pre-processing

ScRNA-seq libraries for 5Ht and 6Ho mice were generated using the 10X Genomics Chromium platform. The resulting gene expression libraries were mapped to the mouse reference genome (mm10, 10x Genomics pre-built reference mm10–2020-A) using Cell Ranger (v.6.1.1). For each scRNA-seq library, we applied quality control (QC) filters on the Cell Ranger filtered matrix to remove low-quality cells, defined as those with ≤ 200 detected features or ≥ 10% of reads mapping to mitochondrial genes. Additionally, cells with unique molecular identifier (UMI) counts ≥ 30,000 were identified as potential doublets and were excluded from further analyses.

Post-QC, the filtered datasets were integrated using the recommended sequential procedures and functionalities described in the Seurat (v.4.4.0) package [23]. The raw gene expression matrices were normalized using the ***SCTransform()*** function. Next, ***SelectIntegrationFeatures()*** was used to select integration features, which were then passed to ***PrepSCTIntegration()*** to prepare the objects for data integration. Integration anchors were identified with ***FindIntegrationAnchors()***, and the datasets were merged using the ***IntegrateData()*** function. After quality control and data integration, the integrated dataset comprised 12,472 cells and 19,140 genes.

### Cell type annotation

#### Clustering-based cell type annotation.

The integrated dataset was then clustered and visualized using the UMAP algorithm based on the top 30 principal components (PCs) computed by PCA. We identified the 20 nearest neighbors using the ***FindNeighbors()*** function and performed clustering using ***FindClusters()*** with a resolution of 0.5. Marker genes for each cluster were then identified by comparing cells in this cluster against all other cells in the same mice using ***FindMarkers*** (test.use = “wilcox”, min.pct = 0.1, logfc.threshold = 0.25, only.pos = TRUE) with a Wilcoxon rank‐sum test. Only genes with a Bonferroni‐adjusted p‐value < 0.05 were considered significant.

Cell type annotation was performed by examining the cluster-specific differentially expressed genes and expression of canonical marker genes. Cells were first classified into four major categories: immune cells (Ptprc), epithelial cells (Epcam, Krt18, and Krt19), endothelial cells (Pecam1), and collagen-producing cells (Col1a2). Each major population was further subdivided into distinct subclusters using an iterative refinement approach, in which cells within each broad category were subset, reprocessed, and re-clustered following the integration and clustering procedures described above.

The epithelial cells were further classified into basal epithelial cells (distinguished by the additional expression of Krt14 and Krt5) and luminal epithelial cells (marked by additional expression of Krt8). The immune cells were further divided into monocytes (Plac8, Lyz2, and Ifitm3), macrophages (Adgre1 and Cd14), neutrophils (S100a8 and S100a9), mast cells (Mcpt4 and Cma1), natural killer (NK) cells (Nkg7, Klrb1c, and Gzmb), B cells (Cd79a, Cd19, and Ms4a1), and T cells (Cd3d, Cd3e, and Cd3g). A distinct subset of immune cells expressing S100a4, Areg, Il7r, Rgcc, Ramp1, Rora, Gata3, Fgl2, Gadd45b, and Ccl1 was classified as type 2 innate lymphoid cells (ILC2s). Dendritic cells (DCs) include classical DC1 (cDC1, identified by Clec9a, Xcr1, and Batf3), classical DC2 (cDC2, defined by Cd209a and Napsa), mature dendritic cells (mDCs, expressing Fscn1, Ccr7, and Il12b), and plasmacytoid dendritic cells (pDCs, marked by Ly6d, Bst2, Ccr9, and Cox6a2).

#### Reference-based cell type annotation.

To improve the robustness of cell type identification and ensure accurate selection of basal epithelial cells without contamination of other cell types (e.g., immune cells, fibroblasts, etc), we cross-validated our clustering-based annotation using a reference-based label transfer method implemented in Seurat [46]. The ***reference dataset*** with annotated cell types was obtained from a published scRNA-seq study of the mouse mammary glands [28].

Specifically, we first created Seurat objects for the ***reference dataset*** and the integrated dataset**,** which served as the ***query dataset***. We then performed filtering to retain high-quality cells (nFeature_RNA > 300, nFeature_RNA < 5000, and percent.mt ≤ 10) in the reference and query datasets and normalized them using ***NormalizeData()***. Highly variable genes in both datasets were identified independently using ***FindVariableFeatures()***.

A shared set of 2,000 integration features was then selected using ***SelectIntegrationFeatures()***, followed by independent scaling and principal component analysis (PCA). We then utilized ***FindIntegrationAnchors()*** and ***IntegrateData()*** to align and integrate the two datasets and remove any batch effects that may arise due to different data sources.

For label transfer, ***FindTransferAnchors()*** was used to identify anchors between the reference and query cells, and ***TransferData()*** projected the reference cell-type labels onto the query cells to generate the cell type annotation for the query cells.

Only basal epithelial cells identified by both the clustering-based cell annotation approach and the reference-based label transfer method were selected and used for network analysis as well as downstream subtype identification and characterization.

### Data preprocessing for downstream analyses

We further processed the annotated integrated dataset for downstream analyses using Scanpy (v.1.9.4) [47] and its pre-processing modules. First, we filtered the cells and the genes in the dataset using the ***filter_genes()*** and ***filter_cells()*** functions. We retained the cells expressing more than 300 and fewer than 5000 genes; genes expressed in at least 5 cells were retained for subsequent analyses. We then normalized total counts to 10,000 per cell using ***normalize_total()*** and natural log-transformed the data to obtain the ***normalized scRNA-seq dataset.***

### Network analysis

Data preparation for network analysisWe removed the genes with the “Rik” suffix (e.g., 5730419F03Rik) and those starting with “Gm” (e.g., Gm31812) from the ***normalized scRNA-seq dataset*** to focus our analysis on well-annotated genes. The dataset contained 12,274 cells and 15321 genes after these pre-processing steps.

Since our analysis focuses on basal epithelial cells, we subset the dataset for these cells to create the ***combined basal dataset*** before performing any subsequent processing. To eliminate any sources of immune cell (particularly macrophage) contamination, we removed all single cells expressing either Ptprc or Adgre1 from the ***combined basal dataset***. After eliminating these cells, the dataset contained 1510 cells and 15321 genes. To further ensure that no contaminating cells were present in the data, we utilized the reference-based method (see the ‘Reference-based cell type annotation’ section above for details) to predict the labels of the basal cells in our data with a reference dataset. Using this approach, we filtered 18 5Ht and 4 6Ho basal cells that were predicted to be other cell types. Applying these filtering steps created the ***filtered combined basal dataset*** that contained 1488 cells and 15321 genes. We then split the data by mouse group to obtain the ***filtered 5Ht basal dataset*** and the ***filtered 6Ho basal dataset*** before subsequent processing.

Then, we performed the subsequent processing steps on the filtered 5Ht, 6Ho, and combined basal datasets separately. We identified highly variable genes (HVGs) using the ***highly_variable_genes()*** function and retained the HVGs with a minimum mean expression of 0.0125, a maximum mean expression of 3.5, and a minimum dispersion of 0.45 for subsequent analyses**.**

To facilitate comparative analysis, we took the union of the HVGs retained in the 5Ht, 6Ho, and the combined basal datasets, creating a set of 4885 genes. Then, we subset the ***filtered combined basal dataset*** to retain these HVGs, yielding the ***pre-processed combined basal dataset*** containing 1488 cells and 4885 genes. The counts were then scaled for each gene to have a unit variance and zero mean.

Finally, the ***pre-processed 5Ht basal dataset***, obtained by subsetting the ***pre-processed combined basal dataset*** for 5Ht cells, contained 972 cells and 4885 genes, while ***the pre-processed 6Ho basal dataset,*** obtained by subsetting the ***pre-processed combined basal dataset*** for 6Ho cells, contained 516 cells and 4885 genes. These datasets were then used for network analysis.

Network analysis using SCENICNetwork analysis was conducted using the SCENIC (v.0.12.1) pipeline in Python, which was applied to the scRNA-seq data from the basal epithelial cells of the 5Ht and 6Ho mice, respectively. The SCENIC workflow involves three main steps: network inference, motif enrichment, and cellular enrichment of regulons.

*Network inference*: SCENIC utilizes the stochastic gradient boosting machine algorithm ***GRNBoost2*** [48] to infer gene regulatory networks based on the co-expression of TFs and their target genes. A set of 1860 TFs obtained from the cisTarget resources database (https://resources.aertslab.org/cistarget) and the ***pre-processed 5Ht and 6Ho basal datasets*** were used, respectively, as input for GRNBoost2. Of these 1860 TFs, 485 TFs were present in the pre-processed datasets and were the basis of our network analysis. The output of this algorithm was an edge list mapping TFs to their target genes with associated importance scores obtained from the regression model.

To account for the stochastic variability of GRNBoost2, we ran the algorithm 20 times for each mouse group, ensuring that the associations between the identified TFs and their target genes were stable and reproducible. For results from each run of the algorithm, we performed motif enrichment as described below.

*Motif enrichment*: The network inferred from above was refined by assessing motif enrichment in the regulatory regions of the identified target genes. This step involved generating modules and regulons. Modules are sets of the identified TFs and their target genes, generated using the ***modules_from_adjacencies()*** function. This function filters the TFs and their target genes by selecting the top-ranked target genes for each identified TF based on their importance scores obtained from the tree-based regression model. These modules were then used to generate regulons through motif enrichment using ***prune2df()*** and ***df2regulons()***.

*Ranking target genes for each identified regulon*: We summarized the results from all 20 runs by calculating summary statistics of the detected target genes for each regulon, including target gene frequencies, importance scores, and the mean, median, and standard deviation of expression levels of the genes. The expression values were calculated across the basal epithelial cells of each mouse group. Target genes were ranked based on the frequency of each gene’s occurrence in the regulon across 20 runs of the SCENIC analysis, with a higher frequency indicating a higher rank. They were then sorted by rank within each regulon.

*Cellular enrichment of regulons*: Regulons detected across all 20 runs were aggregated by the union operation for each mouse group, and their cellular enrichment was estimated by the AUC scores obtained using the ***AUCell()*** function. The output of this step was an AUC matrix, with cells in rows and the detected regulons in columns. A higher AUC score indicates that more target genes of a regulon are actively expressed, reflecting the activity of the TF in regulating gene expression within that specific cell.

### Validation of the identified regulons

The MGI database is a collection of expertly curated information and resources that integrates the Mouse Genome Database (MGD) and the Gene Expression Database (GXD) to support experimental and computational investigations of the laboratory mouse genome. We used the MGI database (v.6.23) to generate a set of TFs active in mouse mammary gland cells. To obtain this set, we first identified all genomic features active in mammary gland cells of mice, which resulted in a set of 23,754 features. We then filtered this set only to include protein-coding genes with DNA-binding transcription factor activity. This resulted in a set of 1032 transcription factors we used for validation.

### Cluster analysis and subcluster alignment

To identify subclusters of basal epithelial cells in 5Ht and 6Ho mice, we first standardized the AUC matrices so that each column (representing a regulon) has a mean of 0 and a standard deviation of 1. Then, we applied agglomerative, average-linkage hierarchical clustering on the standardized AUC matrices to partition the regulons and the cells into distinctive groups based on the cellular activities of regulons. Correlation was used to measure the similarity between variables (regulons or cells). The hierarchical clustering algorithm was implemented using Cluster 3.0 (v.1.59).

Specifically, clustering was first performed on the basal epithelial cells and the regulons in the 5Ht mice. To facilitate the alignment of the subclusters in the 5Ht and 6Ho mice, we reordered the regulons in the 6Ho mice to match the order of the clustered 5Ht regulons. We then performed clustering on the basal epithelial cells of the 6Ho mice without clustering the regulons. This strategy facilitated comparison between the two mouse groups by maintaining the same regulon order.

The subclusters in the two mouse groups were then aligned based on discernible patterns, as revealed in the heatmaps of the clustered AUC matrices. These subclusters were categorized as either “similar” (exhibiting comparable patterns in both mouse groups) or “unique” (displaying a pattern specific to only one group).

### Visualization

The UMAP plots were generated using the ***DimPlot()*** function in Seurat to illustrate the spatial distributions of the identified subclusters in two‑dimensional (2D) space. The ***FeaturePlot()*** function was used to visualize expression levels of genes across all basal epithelial cells in the 5Ht or 6Ho mice. Dot plots were generated using the ***dotplot()*** function from Scanpy. For heatmaps, we utilized TreeView (v.1.2.0) (https://sourceforge.net/projects/jtreeview/files/jtreeview/1.2.0/) to display the hierarchical clustering of the AUC matrices in the two mouse groups.

### Centroid analysis of subclusters

AUC matrices for the 5Ht and 6Ho mice were first combined to create a single matrix containing cells as rows and regulons as columns. The matrix was then scaled column-wise, so that each column has a mean of 0 and a standard deviation of 1. We then applied a principal component (PC) analysis to the scaled matrix to select top 15 PCs; after that, UMAP was performed using the ***umap package*** (v.0.2.10.0) (n_neighbors = 30, min_dist = 0.3, Euclidean metric) in R to obtain a 2D embedding of the AUC regulon scores for each cell. Cells were then annotated on the UMAP plots by subclusters (S1–S3, U1_wt) from each mouse group to illustrate the cross‑group alignment of the subclusters.

For quantitative comparison of the distances of the subclusters, we first computed the centroid of each subcluster as the mean of the 2D UMAP embeddings of all the cells within the subcluster, we then calculated the pairwise Euclidean distances between the centroids of the subclusters of the 5Ht and 6Ho mice to measure the distances of the subclusters in the two mouse groups.

### Differential gene expression analysis

We performed DGE analysis using the ***normalized scRNA-seq dataset*** to identify DEGs in all basal epithelial subclusters identified in both mouse groups. DEGs were identified using the ***FindMarkers()*** function with the MAST test and an absolute log_2_ fold change threshold of 0.585 (i.e., a fold change of 1.5 in the original scaling). We also set the minimum difference in the fraction of detection (“min.diff.pct”) between the two compared groups and the “min.pct” to be 0.15, respectively. We used the MAST test because it identifies DEGs between two groups of cells using a hurdle model tailored to scRNA-seq data [49]. ***FindMarkers()*** used the Bonferroni correction to account for multiple hypothesis testing.

For the “similar” subclusters (S1–S3), DEGs were identified between each subcluster and its counterpart in the two mouse groups. For the “unique” subcluster (i.e., U1_wt of the 5Ht mice), DEGs were identified between the “unique” subcluster and all the other basal epithelial cells of the same mouse group.

### Identification of significantly different regulons between the 5Ht and 6Ho mice

For each subcluster of basal epithelial cells, we identified regulons with significantly different AUC scores between the two mouse groups using the Wilcoxon rank-sum test, followed by the multiple testing correction using the Benjamini-Hochberg procedure to control the false discovery rate (FDR) [50]. Regulons with an FDR-adjusted p-value < 0.05 and an absolute mean AUC score difference > 0.15 were considered significantly different. For the “similar” subclusters (S1–S3), the AUC scores of each regulon were compared between corresponding basal subclusters in the two mouse groups. For the “unique” subclusters (i.e., U1_wt of the 5Ht mice), the AUC scores were compared between cells in each “unique” subcluster and all other basal epithelial cells of the same mouse group.

### Functional enrichment analysis

To identify functional groups of genes significantly enriched among the DEGs from the DGE analysis and the DETGs in the detected regulons from the network analysis, we performed functional enrichment analysis using the ***enrichGO()*** and ***enrichKEGG()*** functions from the ***clusterProfiler*** package in R [51]. These functions estimate overrepresentation in Gene Ontology (GO) and KEGG pathways among genes of interest using the hypergeometric test. The set of 18,103 unique genes used in the DGE analysis was employed as the gene universe for the enrichment analysis of the DEGs. The set of 4885 unique genes used for the network analysis was employed as the gene universe for the DETGs. A GO term or a KEGG pathway was considered significantly enriched if it had a Benjamini-Hochberg FDR-adjusted p-value < 0.05.

### External validation of results

To validate our results obtained from the U1_wt basal subcluster of the control mice, we investigated two publicly available external microarray datasets, GSE65194 [33] and GSE45827 [34] in the GEO database, collected from the same cohort of breast cancer patients using Affymetrix U133 Plus 2.0 microarrays. Specifically, the GSE65194 dataset contains 55 arrays from 41 TNBC samples, and 11 arrays from as many normal tissues. Of the 55 TNBC arrays, technical duplicates were also generated for 14 samples. This dataset was processed using a custom Affymetrix chip definition file (CDF) to improve the interpretation of the GeneChip probe sets and enhance the accuracy of downstream analyses. The GSE45827 dataset includes microarray data from the same 41 TNBC and 11 normal tissue samples, each with one technical replicate, processed using the default probe set definitions. The DGE analysis for both datasets was performed with the GEO2R tool, which carries out quantile normalization and fits gene-wise linear models using the ***limma*** package [52] in R, followed by empirical-Bayes moderation to find DEGs between the 41 TNBC and 11 normal tissues. DEGs were considered significant if the Benjamini–Hochberg FDR-adjusted p‑value < 0.05 and absolute log₂FC > 0.585 (i.e., a fold change of 1.5 in the original scaling). Then we compared these two DEG lists against the genes identified in our subcluster analysis. Because of the differences in array composition, replicate structure, and CDF annotations, these two microarray datasets yield different DEG results, and their concordant DEG overlaps provide robust cross‐validation of our findings.

### Immunohistochemistry analysis of the mammary glands in 5Ht and 6Ho mice

To identify macrophages, mammary gland sections were immunostained using F4/80 antibodies, a marker known to label macrophages. The sections were deparaffinized and rehydrated through a graded alcohol series (100%, 95%, and 70%) into phosphate-buffered saline (PBS) to remove paraffin and restore the tissue to an aqueous state suitable for staining. Endogenous peroxidase activity was blocked by incubation with 3% H₂O₂ for 20 minutes to prevent non-specific background staining. For antigen retrieval, slides were steamed in citrate sodium buffer for 30 minutes to expose epitopes and enhance antibody binding. The sections were then blocked with an Avidin/Biotin Blocking Kit (Vector Laboratories, SP-2001) and 5% goat serum to prevent the non-specific binding of antibodies to biotin or other proteins.

After blocking, the tissue was incubated with primary anti-F4/80 antibodies at 4°C overnight to allow specific binding to macrophage antigens. The sections were treated with Biotinylated Goat Anti-Rabbit IgG Antibody (Vector Laboratories, BA-1000) at room temperature for 60 minutes, followed by incubation with Streptavidin-Horseradish Peroxidase (Vector Laboratories, SA-5704) for 30 minutes to amplify the staining signal. The staining signal was developed using diaminobenzidine (DAB, Vector Laboratories, SK-4100), producing dark brown staining to mark macrophages. The sections were then counterstained with hematoxylin to visualize cell nuclei, and images were captured using a Nikon Eclipse 80i microscope.

## Supporting information

S1 Fig Immunofluorescence images showing that P4HA1 expression in 6Ho knockout (KO) mice was silenced in K14-positive mammary epithelial cells. (TIF)

S2 Fig Integrated UMAP plots split by the (A) 5Ht, and (B) 6Ho mouse groups. (TIF)

S3 Fig Expression of canonical marker genes in basal and luminal epithelial cells, and macrophages identified in 5Ht and 6Ho mice. Dot plots show expression of marker genes for epithelial basal cells (Epcam, Krt18, Krt19, Krt14, Krt5), epithelial luminal cells (Epcam, Krt18, Krt19, Krt8), macrophages (Cd14, Adgre1), and immune cells (Ptprc) across the three major cell types in the 5Ht and 6Ho mice.
(TIF)

S4 Fig Subclusters of the 5Ht and 6Ho mice revealed by hierarchical clustering with dendrograms. Heatmap showing the dendrograms of the basal epithelial subclusters and their regulon patterns in the (A) 5Ht mice, and (B) 6Ho mice. Cells are shown in rows; the 245 common regulons detected in both mouse groups are shown in columns.
(TIF)

S5 Fig Expression of canonical marker genes in basal epithelial subclusters of the 5Ht and 6Ho mice. Dot plots show expression of epithelial basal markers (Epcam, Krt18, Krt19, Krt14, Krt5), epithelial luminal markers (Epcam, Krt18, Krt19, Krt8), macrophage markers (Cd14, Adgre1), and immune cell markers (Ptprc) across all basal epithelial subclusters in the 5Ht and 6Ho mice.
(TIF)

S6 Fig UMAP plots show subclusters of the basal epithelial cells in the 5Ht and 6Ho mice based on their AUC scores of the regulon activities. (TIF)

S7 Fig UMAP plots showing all subclusters of the basal epithelial cells in the 5Ht and 6Ho mice based on their scRNA-seq expression profiles. The subclusters were identified based on activities of the common regulons in the 5Ht (left panel) and 6Ho (middle panel) basal epithelial cells. Integrated plots illustrating “similar” subclusters from both the mouse groups are in the right panel.
(TIF)

S8 Fig Four inflammatory signature genes reported in a myoepithelial subcluster (Epi-C11) of the mouse aging atlas study were also upregulated in the 5Ht U1_wt subcluster. Dot plots show 4 pro-inflammatory genes, Cd74, H2-Ab1, H2-Eb1, and Ccl2, upregulated in subcluster U1_wt of the 5Ht mice.
(TIF)

S1 Table Numbers of single cells for three major cell types in the 5Ht and 6Ho mice. (PDF)

S2 Table Transcription factors (TFs) detected in mammary basal epithelial cells of the 5Ht and 6Ho mice. These TFs in (A) 5Ht and (B) 6Ho mice were validated using the MGI database.
(PDF)

S3 Table Numbers of single cells, differentially expressed genes (DEGs) and significantly different regulons in the basal epithelial subclusters of the 5Ht and 6Ho mice. (PDF)

S4 Table Pairwise distances between the centroids of the basal epithelial subclusters in 5Ht and 6Ho mice. (PDF)

S5 Table Enriched functional groups of genes among DEGs between the “similar” subclusters of the 5Ht and 6Ho mice. (PDF)

S6 Table Enriched functional groups of genes among DEGs in subcluster U1_wt of the 5Ht mice. (PDF)

S7 Table Significantly different regulons and the enriched functional groups of genes among their DETGs in subcluster S3 of the 5Ht and 6Ho mice. (PDF)

S8 Table Significantly different regulons and the enriched functional groups of genes involved in collagen metabolic process and stem cell differentiation among their DETGs in subcluster U1_wt of the 5Ht mice. (PDF)

S9 Table Significantly different regulons and the enriched functional groups of genes among their DETGs in subcluster U1_wt of the 5Ht mice. (PDF)

S10 Table Significantly different regulon TFs in 5Ht subcluster U1_wt showed open chromatin peaks and expressed in 3M mice in a recent mouse aging atlas study. (XLSX)

S11 Table DEGs in 5Ht subcluster U1_wt showed open chromatin peaks, expressed in 3M mice, and were also DEGs in subcluster Epi-C11 in a recent mouse aging atlas study. (XLSX)

S12 Table Significantly different regulon TFs and DEGs in subcluster U1_wt of the 5Ht mice are differentially expressed between human TNBC and normal samples from the external microarray datasets. (XLSX)
