# Supplementary figures and images for: Single-cell analysis of gene regulatory networks in the mammary glands of P4HA1-knockout mice
Source: PLoS Genet. 2025 Jul 22;21(7):e1011505. doi: 10.1371/journal.pgen.1011505 (PMC12310035; doi:10.1371/journal.pgen.1011505)

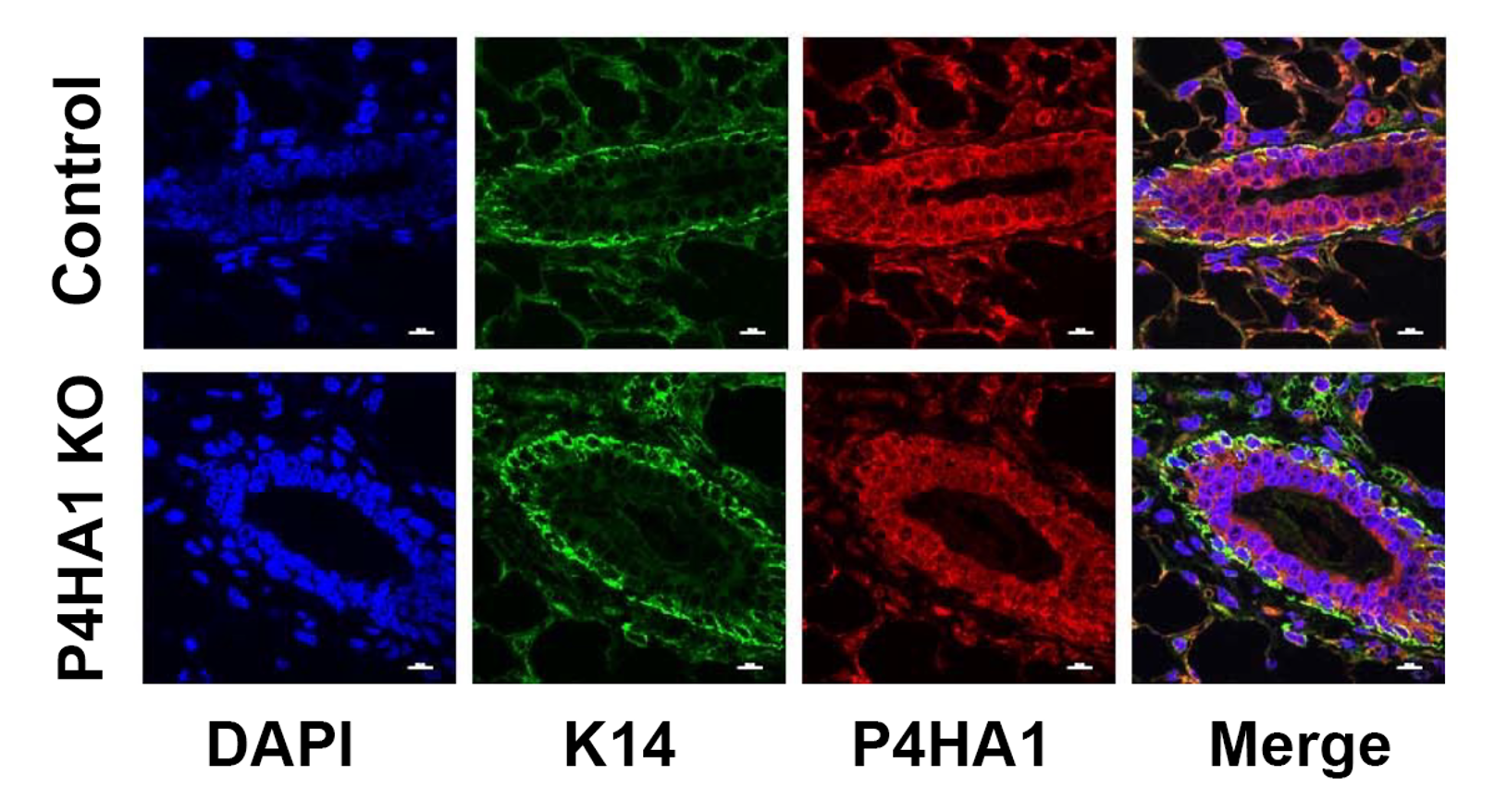

Supplement: S1 Fig — (TIF) [file pgen.1011505.s001.tif]

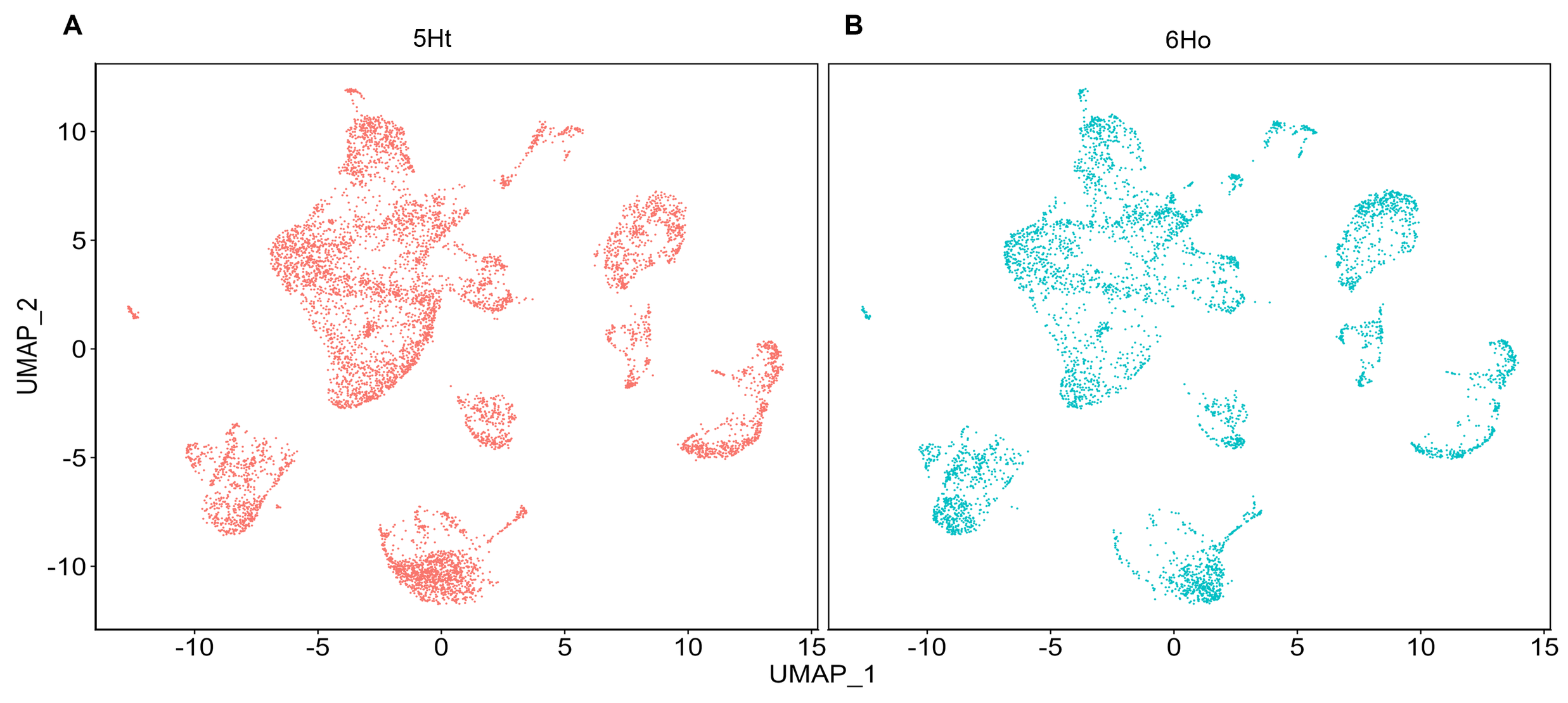

Supplement: S2 Fig — (TIF) [file pgen.1011505.s002.tif]

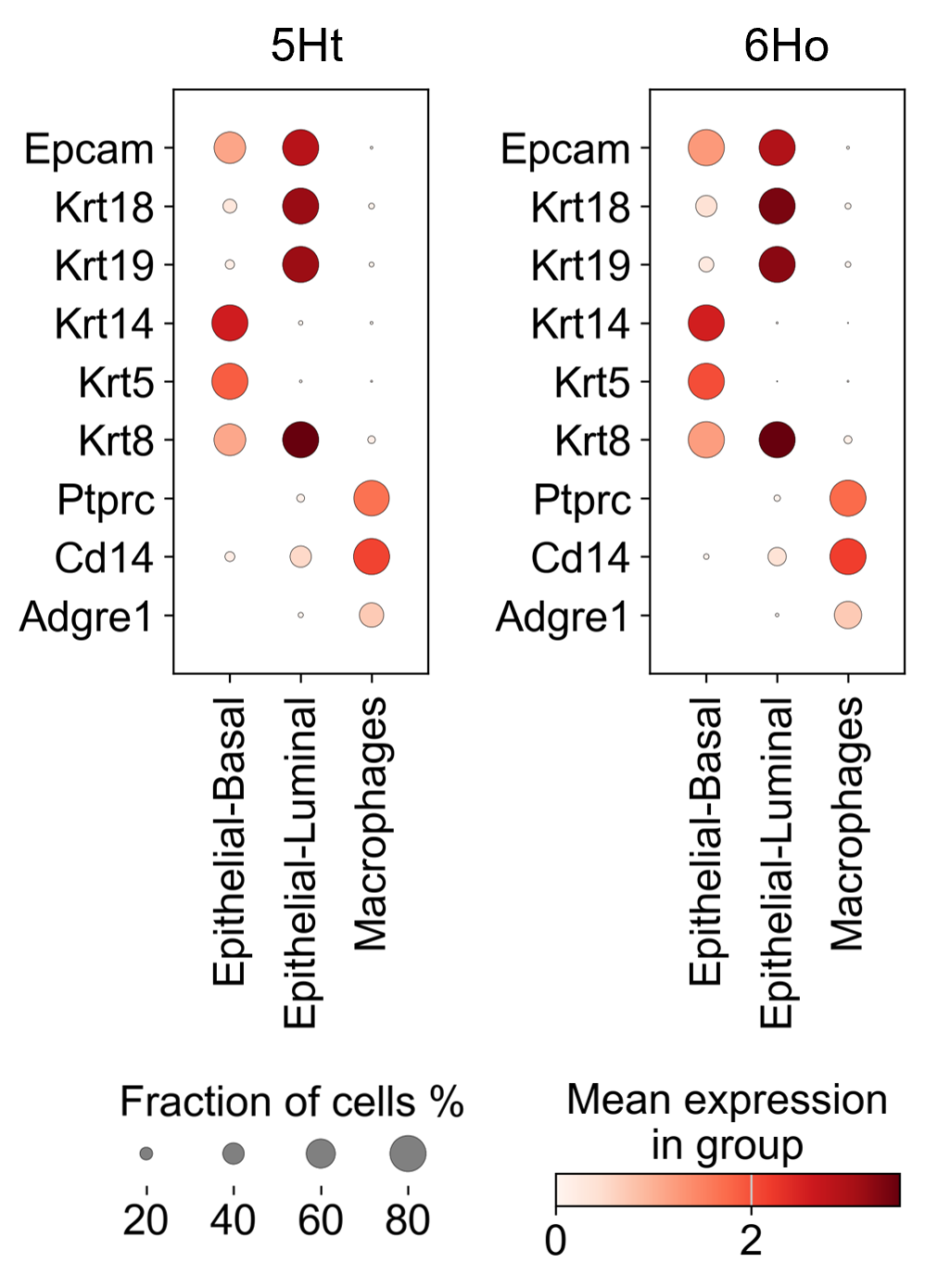

Supplement: S3 Fig — Dot plots show expression of marker genes for epithelial basal cells (Epcam, Krt18, Krt19, Krt14, Krt5), epithelial luminal cells (Epcam, Krt18, Krt19, Krt8), macrophages (Cd14, Adgre1), and immune cells (Ptprc) across the three major cell types in the 5Ht and 6Ho mice. (TIF) [file pgen.1011505.s003.tif]

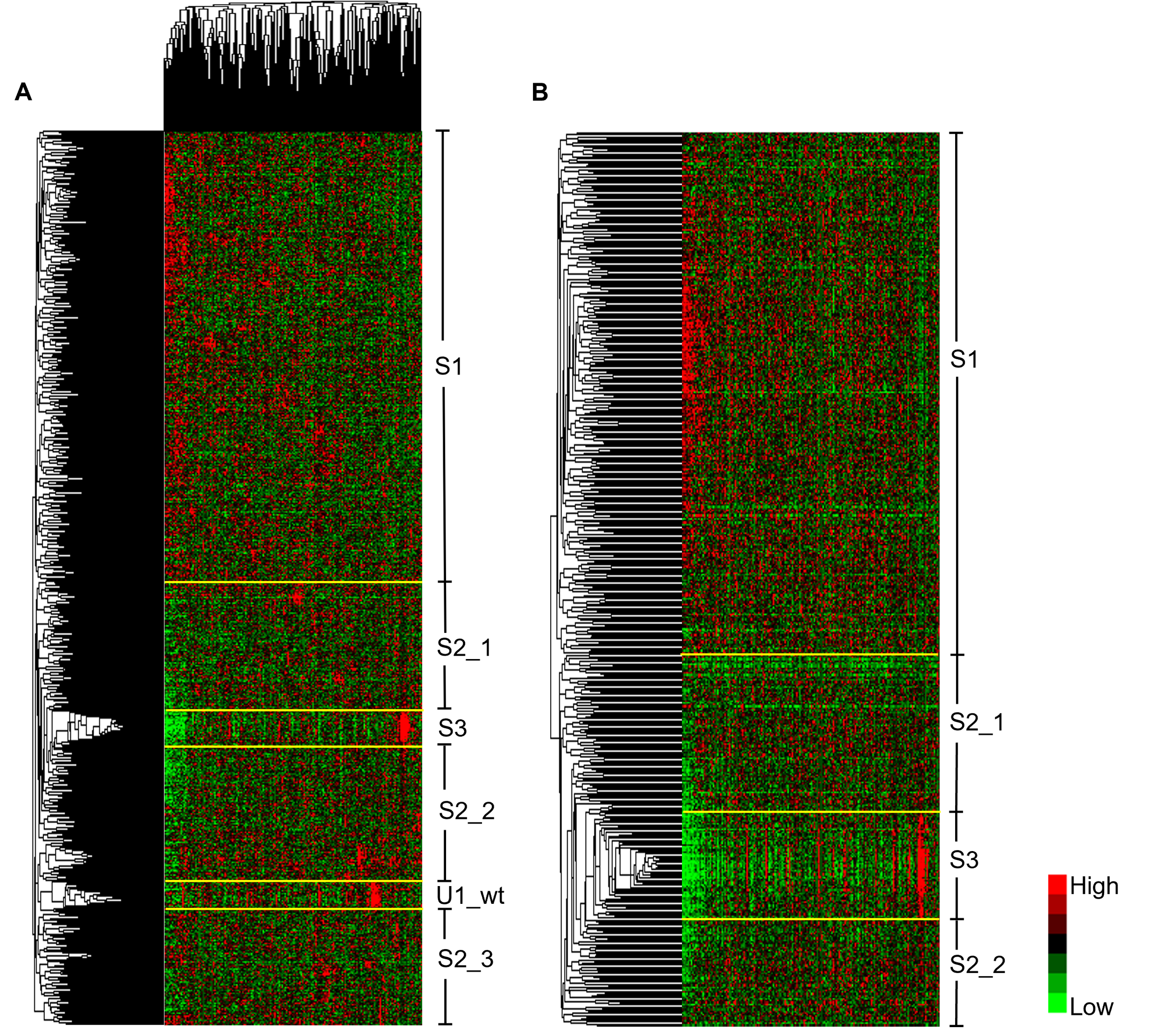

Supplement: S4 Fig — Heatmap showing the dendrograms of the basal epithelial subclusters and their regulon patterns in the (A) 5Ht mice, and (B) 6Ho mice. Cells are shown in rows; the 245 common regulons detected in both mouse groups are shown in columns. (TIF) [file pgen.1011505.s004.tif]

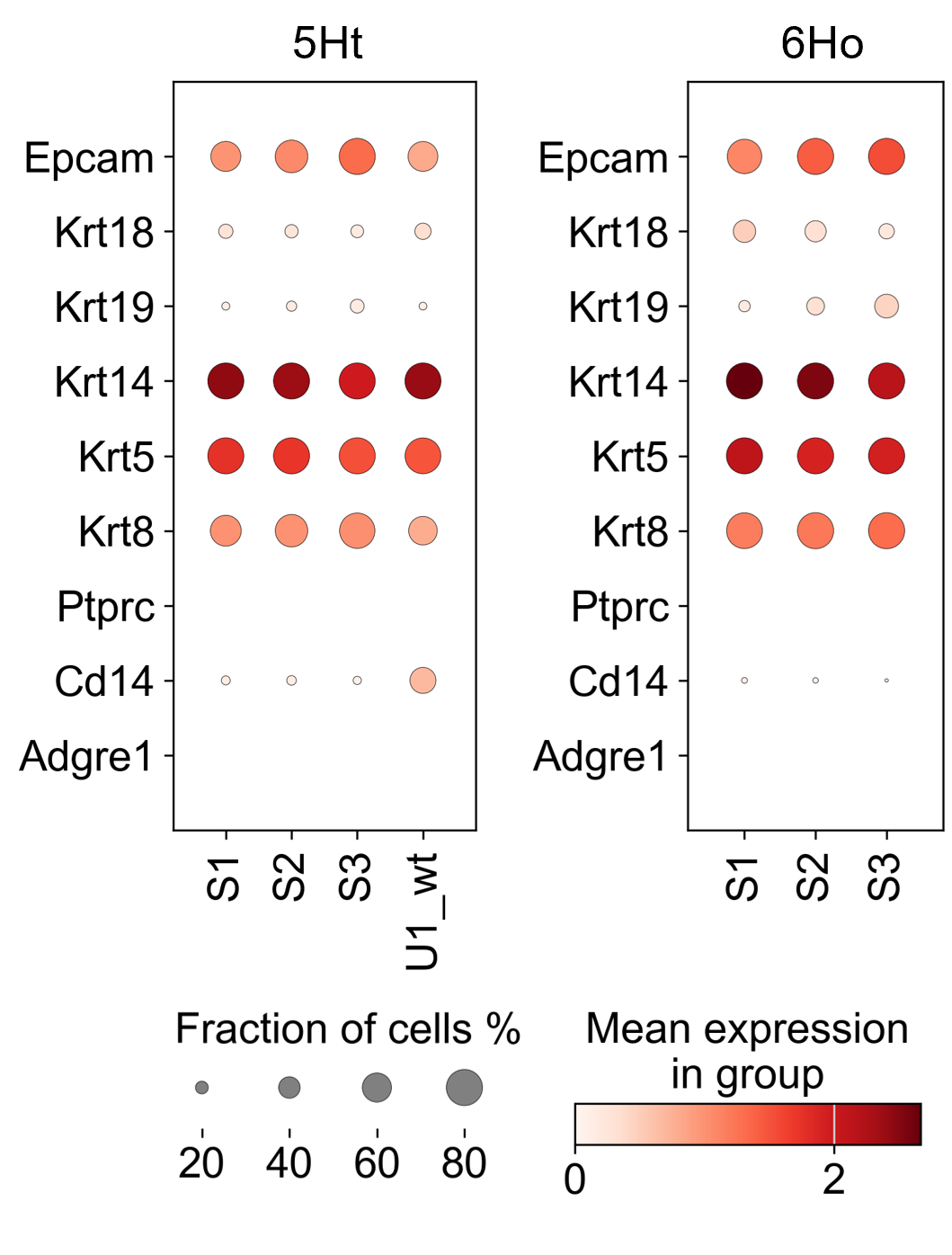

Supplement: S5 Fig — Dot plots show expression of epithelial basal markers (Epcam, Krt18, Krt19, Krt14, Krt5), epithelial luminal markers (Epcam, Krt18, Krt19, Krt8), macrophage markers (Cd14, Adgre1), and immune cell markers (Ptprc) across all basal epithelial subclusters in the 5Ht and 6Ho mice. (TIF) [file pgen.1011505.s005.tif]

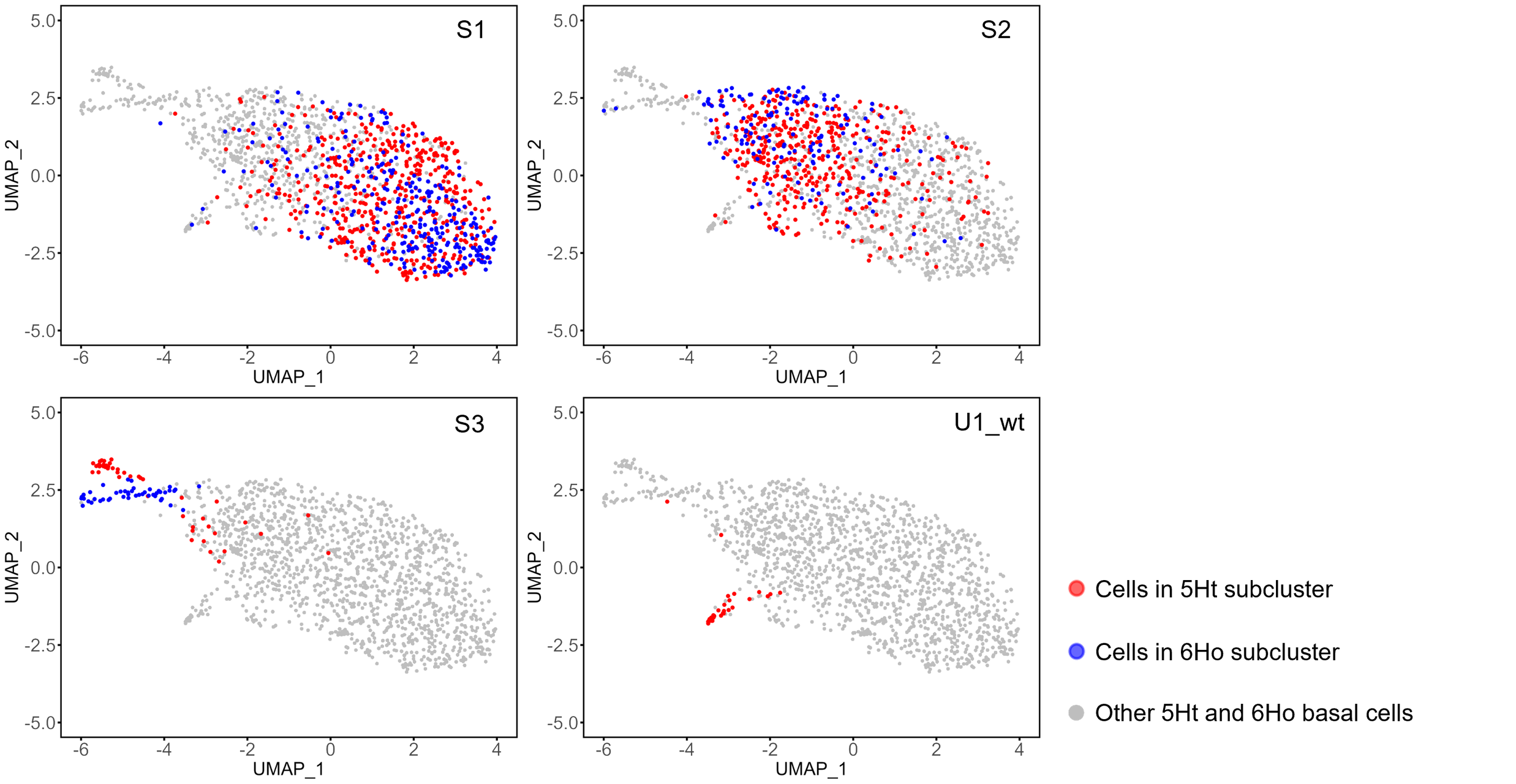

Supplement: S6 Fig — (TIF) [file pgen.1011505.s006.tif]

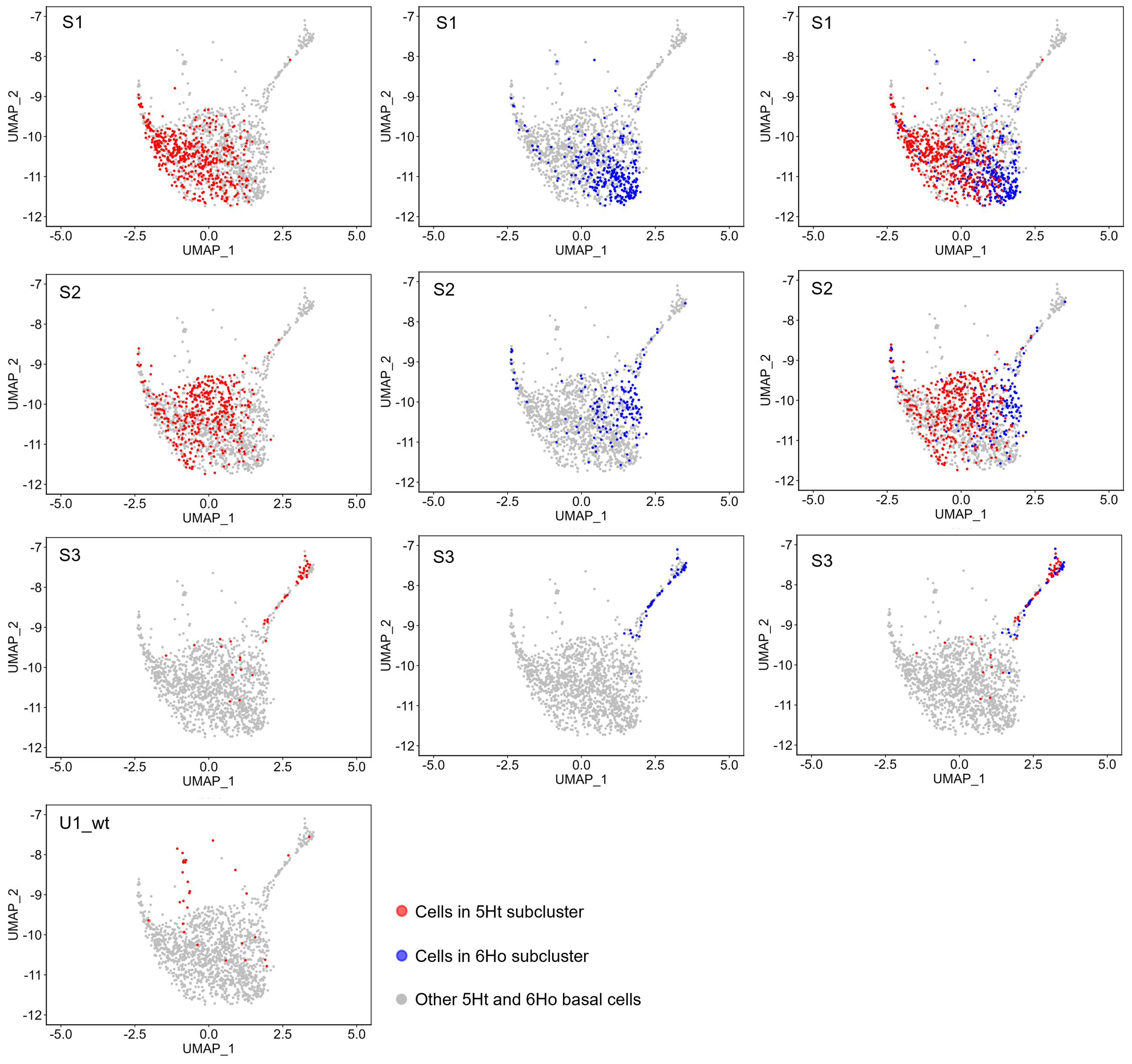

Supplement: S7 Fig — The subclusters were identified based on activities of the common regulons in the 5Ht (left panel) and 6Ho (middle panel) basal epithelial cells. Integrated plots illustrating “similar” subclusters from both the mouse groups are in the right panel. (TIF) [file pgen.1011505.s007.tif]

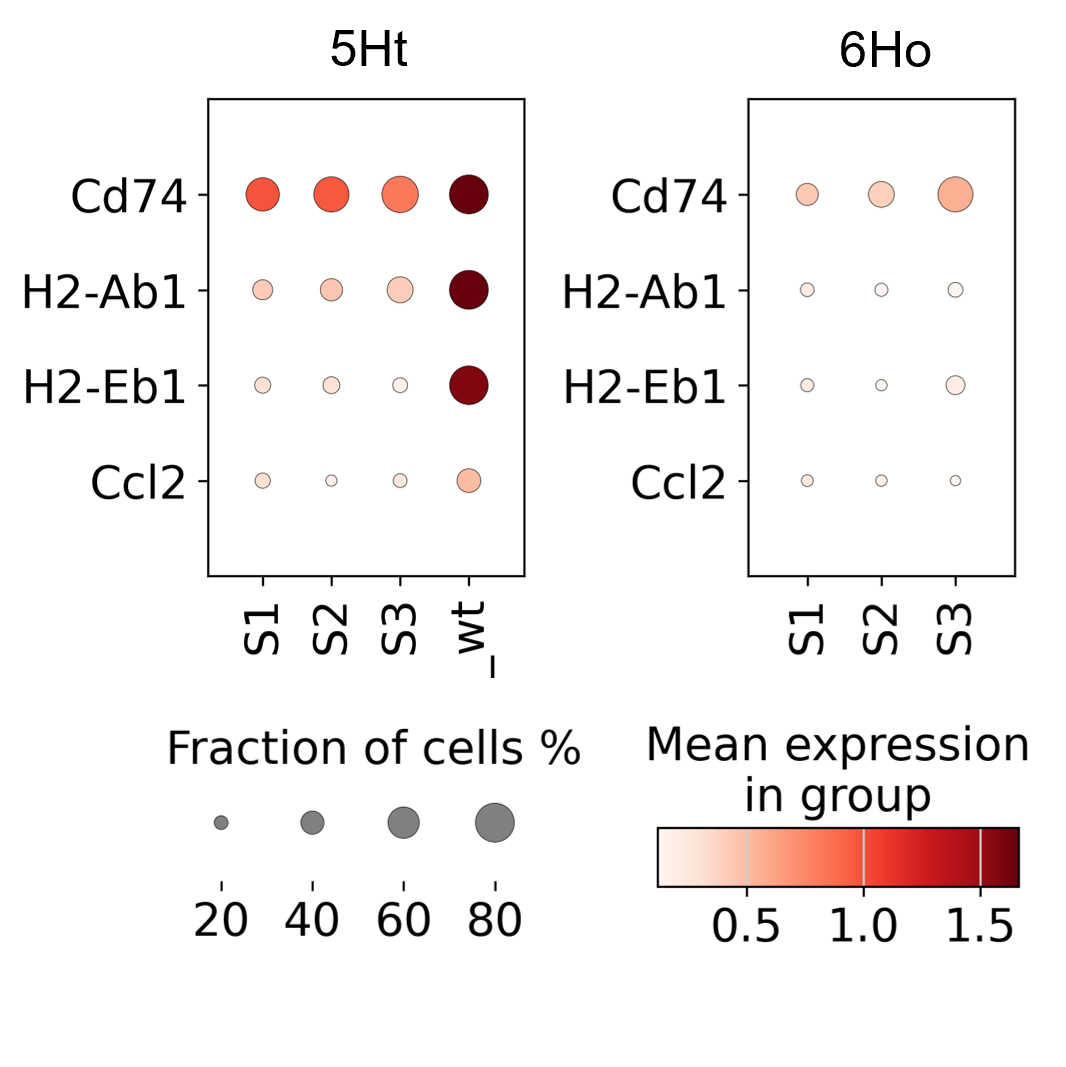

Supplement: S8 Fig — Dot plots show 4 pro-inflammatory genes, Cd74, H2-Ab1, H2-Eb1, and Ccl2, upregulated in subcluster U1_wt of the 5Ht mice. (TIF) [file pgen.1011505.s008.tif]
