# Supplementary material for: Single-cell analysis of gene regulatory networks in the mammary glands of P4HA1-knockout mice
Source: PLoS Genet. 2025 Jul 22;21(7):e1011505. doi: 10.1371/journal.pgen.1011505 (PMC12310035; doi:10.1371/journal.pgen.1011505)
Supplement: S1 Table — (PDF) [file pgen.1011505.s009.pdf]

S1 Table: Numbers of single cells for three major cell types in the 5Ht and 6Ho mice.

	Number of basal epithelial cells	Number of luminal epithelial cells	Number of macrophages
5Ht	972	917	1290
6Ho	516	733	673
