# Supplementary material for: Single-cell analysis of gene regulatory networks in the mammary glands of P4HA1-knockout mice
Source: PLoS Genet. 2025 Jul 22;21(7):e1011505. doi: 10.1371/journal.pgen.1011505 (PMC12310035; doi:10.1371/journal.pgen.1011505)
Supplement: S2 Table — These TFs in (A) 5Ht and (B) 6Ho mice were validated using the MGI database. (PDF) [file pgen.1011505.s010.pdf]

S2 Table: Transcription factors (TFs) detected in mammary basal epithelial cells of the 5Ht and 6Ho mice. These TFs in (A) 5Ht and (B) 6Ho mice were validated using the MGI database.

(A) 5Ht mice

	Number of detected TFs	Number of overlapping TFs detected in this study and in the MGI database (%)
All TFs in 5Ht	289	242 (83.7%)
Common TFs in 5Ht and 6Ho	245	215 (87.7%)
Unique TFs in 5Ht	44	27 (61.3%)

Note: % represents the percentage of overlap, calculated by dividing the "number of overlapping TFs detected in this study and the MGI database" listed in column 3, by the total "number of detected TFs" in column 2.

(B) 6Ho mice

	Number of detected TFs	Number of overlapping TFs detected in this study and the MGI database (%)
All TFs in 6Ho	290	244 (84.1%)
Common TFs in 5Ht and 6Ho	245	215 (87.7%)
Unique TFs in 6Ho	45	29 (64.4%)

Note: see legend in (A).
