# Supplementary material for: Single-cell analysis of gene regulatory networks in the mammary glands of P4HA1-knockout mice
Source: PLoS Genet. 2025 Jul 22;21(7):e1011505. doi: 10.1371/journal.pgen.1011505 (PMC12310035; doi:10.1371/journal.pgen.1011505)
Supplement: S3 Table — (PDF) [file pgen.1011505.s011.pdf]

S3 Table: Numbers of single cells, differentially expressed genes (DEGs) and significantly different regulons in the basal epithelial subclusters of the 5Ht and 6Ho mice.

(A) Numbers of single cells in the subclusters of the 5Ht and 6Ho mice.

Subcluster	# of single cells in 5Ht (%)	# of single cells in 6Ho (%)
S1	489 (50.3%)	301 (58.3%)
S2	409 (42.2%)	156 (30.3%)
S3	45 (4.6%)	59 (11.4%)
U1_wt	29 (2.9%)	

(B) Numbers of DEGs and significantly different regulons in the subclusters of the 5Ht and 6Ho mice.

Subcluster	# of DEGs	# of significantly different regulons
S1	479	47
S2	1107	34
S3	1647	99
U1_wt	534	81
