# Supplementary material for: Single-cell analysis of gene regulatory networks in the mammary glands of P4HA1-knockout mice
Source: PLoS Genet. 2025 Jul 22;21(7):e1011505. doi: 10.1371/journal.pgen.1011505 (PMC12310035; doi:10.1371/journal.pgen.1011505)
Supplement: S4 Table — (PDF) [file pgen.1011505.s012.pdf]

S4 Table: Pairwise distances between the centroids of the basal epithelial subclusters in 5Ht and 6Ho mice.

Subcluster	S1_6Ho	S2_6Ho	S3_6Ho
S1_5Ht	**0.56**	3.28	7.01
S2_5Ht	2.97	**0.92**	4.59
S3_5Ht	6.87	3.09	**0.83**
U1_wt_5Ht	4.77	2.95	3.99

Note: The centroids of the subclusters were calculated using the two-dimensional UMAP embeddings of the cells in each subcluster derived from the AUC scores of the regulon activities (see Materials and Methods for details).
