# Supplementary material for: Single-cell analysis of gene regulatory networks in the mammary glands of P4HA1-knockout mice
Source: PLoS Genet. 2025 Jul 22;21(7):e1011505. doi: 10.1371/journal.pgen.1011505 (PMC12310035; doi:10.1371/journal.pgen.1011505)
Supplement: S5 Table — (PDF) [file pgen.1011505.s013.pdf]

S5 Table: Enriched functional groups of genes among DEGs between the “similar” subclusters of the 5Ht and 6Ho mice.

(A) Subcluster S1

Enriched GO/KEGG Terms	DEGs	# of DEGs	# of DEGs upregulated in 6Ho	Adjusted p-val
Regulation of Protein Stability	Cd74, Dad1, Tbrg1, Pdcl3, Bag3, Rad23a, Phb2, Stub1, Hspa1a, Tardbp, Fbxw7, Ddost, Tbx3, Gapdh, Pin1, Commd1, Glmp, Hspa1b, Park7, Src, Tbl1x, Pik3r1, Plpp3, Derl1, Golga7, Cct3, Pink1	27	26	1.3E-05
Response to unfolded protein	Hspa2, Atf3, Bag3, Stub1, Dab2ip, Bax, Hspa1a, Serp1, Hspb1, Hspa1b, Ube2j2, Pik3r1, Derl1, Herpud1, Nck2	15	15	1.0E-04
Chaperone Mediated Protein Folding	Cd74, Hspa2, Pdcl3, Hspa1a, Fkbp11, Hspb1, Hspa1b, Hspe1, Ppid, Cct3	10	9	6.0E-04
Cellular Respiration	Ndufs7, Cisd1, Ndufa12, Ndufa8, Iscu, Ndufa6, Ndufv2, Ndufb10, Ndufb7, Ndufab1, Prelid1, Uqcrfs1, Mrps36, Cox7a2l, Arl2, Suclg1, Atp5d, Bax, Cycs, Ndufs3, Ndufb5, Ndufs2, Ndufv1, Cox5a, Ndufb6, Ndufb3, Ndufa5, Ndufb8, Ndufa9, Ndufs8, Cox5b, Atp5j, Atp5c1, Idh2, Sdhb, Mdh2, Park7, Pink1	38	38	9.2E-17
Oxidative Phosphorylation	Ndufs7, Ndufa12, Ndufa8, Iscu, Ndufa6, Ndufv2, Ndufb10, Ndufb7, Ndufab1, Uqcrfs1, Cox7a2l, Atp5d, Cycs, Ndufs3, Ndufb5, Ndufs2, Ndufv1, Cox5a, Ndufb6, Ndufb3, Ndufa5, Ndufb8, Ndufa9, Ndufs8, Cox5b, Atp5j, Atp5c1, Sdhb, Park7, Pink1	30	30	4.5E-17
Glycolysis	Akr1a1, Aldh7a1, Pkm, Tpi1, Ldhb, Gapdh, Pfkl, Adh5	8	8	1.6E-02
Mitochondrial Respiratory Chain Complex Assembly	Ndufs7, Ndufa12, Ndufa8, Ndufa6, Ndufb10, Ndufb7, Ndufab1, Ndufs3, Ndufb5, Ndufs2, Ndufb6, Ndufb3, Ndufa5, Ndufb8, Ndufa9, Ndufs8, Cox14, Tmem223	18	18	3.1E-09

(B) Subcluster S2

Enriched GO/KEGG Terms	DEGs	# of DEGs	# of DEGs upregulated in 6Ho	Adjusted p-val
Response to unfolded protein	Stub1, Tmem33, Pdia6, Bok, Hspd1, Manf, Selenos, Dnajc3, Ccnd1, Bax, Amfr, Jkamp, Hspa1b, Atf6b, Atf3, Ube2j2, Dnajb9, Bak1	18	18	2.0E-02
Chaperone Mediated Protein Folding	Hspe1, Cd74, Pdcl3, Fkbp4, Fkbp2, Ppid, Unc45a, Cct5, Hspa1b, Cct7, Cct3, Sgta, Cct8, Cct4, Fkbp11	15	14	5.0E-04

Cellular Respiration	Ndufv1, Mdh2, Idh3g, Cox5a, Suclg1, Atp5j, Cisd1, Cyc1, Ndufb3, Ndufv2, Ndufs7, Ndufs3, Uqcrc1, Ndufb7, Ndufa9, Mtch2, Ndufb6, Mybbp1a, Cs, Stoml2, Cycs, Sdhb, Uqcrfs1, Dnajc15, Uqcrq, Ndufa8, Ndufab1, Ndufs4, Uqcc2, Ndufs8, Ndufa12, Mdh1, Iscu, Ndufs5, Trap1, Ide, Atp5d, Ndufc2, Ndufa11, Ndufb10, Idh2, Ndufv3, Sdhc, Ndufa13, Dld, Cox7a2, Ndufb8, Chchd4, Akt1, Ndufa2, Ndufs6, Bax, Uqcr10, Ndufb5, Sdhd, Atp5j2, Ndufb9, Gadd45gip1, Ndufb4, Uqcrb, Ndufa5, Mrps36, Arl2, Cox5b, Park7, Atp5o, Adsl, Uqcrc2, Fh1, Pink1, Atp5c1, Prelid1, Cox6a1	73	73	6.9E-30
Oxidative Phosphorylation	Ndufv1, Cox5a, Atp5j, Cyc1, Ndufb3, Ndufv2, Ndufs7, Ndufs3, Uqcrc1, Ndufb7, Ndufa9, Mtch2, Ndufb6, Stoml2, Cycs, Sdhb, Uqcrfs1, Dnajc15, Uqcrq, Ndufa8, Ndufab1, Ndufs4, Uqcc2, Ndufs8, Ndufa12, Iscu, Ndufs5, Atp5d, Ndufc2, Ndufa11, Ndufb10, Ndufv3, Sdhc, Ndufa13, Dld, Cox7a2, Ndufb8, Ndufa2, Ndufs6, Uqcr10, Ndufb5, Sdhd, Atp5j2, Ndufb9, Gadd45gip1, Ndufb4, Uqcrb, Ndufa5, Cox5b, Park7, Atp5o, Uqcrc2, Pink1, Atp5c1, Cox6a1	55	55	1.1E-29
Mitochondrial Respiratory Chain Complex Assembly	Ndufb3, Ndufs7, Ndufs3, Ndufb7, Ndufa9, Ndufb6, Ndufa8, Ndufab1, Ndufs4, Uqcc2, Ndufs8, Ndufa12, Ndufs5, Ndufc2, Ndufa11, Ndufb10, Ndufa13, Ndufb8, Chchd4, Ndufaf2, Ndufa2, Ndufs6, Ndufb5, Ndufb9, Ndufb4, Tmem126a, Ndufa5, Coa3, Tfam	29	29	7.9E-12
Response to Endoplasmic Reticulum Stress	Stub1, Sec61b, Tmem33, Pdia3, Calr, Pdia6, Gorasp2, Ubxn1, Sel1l, Bok, Manf, Ubqln1, Selenos, Dnajc3, Rnf121, Ccnd1, Bax, Amfr, Jkamp, Uba5, Erp29, Canx, Sgta, Bcap31, Atf6b, Atf3, Park7, Ube2j2, Dnajb9, Aup1, Opa1, Bak1	32	32	8.6E-03

(C) Subcluster S3

Enriched GO/KEGG Terms	DEGs	# of DEGs	# of DEGs upregulated in 6Ho	Adjusted p-val
Response to Misfolded Protein	Rnf126, Stub1, Sdf2l1, Derl1, Rnf5, Akirin2, Tor1a, Dnajb14, Dnajb12	9	9	1.5E-02
Chaperone Mediated Protein Folding	Cct3, Cct8, Fkbp4, Ppid, Dnajc5, Sgta, Cct7, Cct4, Hspa9, Sdf2l1, Tcp1, Fkbp2, Tor1b, Fkbp5, Tor1a, Tor2a, Dnajb14, Dnajb12	18	18	1.1E-03
Cellular Respiration	Cycs, Ndufa9, Cox5b, Sdhb, Ndufs8, Atp5d, Uqcrc1, Fh1, Mybbp1a, Sdha, Ndufa8, Mdh1, Idh3b, Iscu, Cyc1, Gadd45gip1, Ndufab1, Cox6a1, Trap1, Ndufb6, Ndufb8, Uqcc2, Mrps36, Ndufs5, Sdhc, Slc25a13, Mdh2,	56	56	3.7E-09

	Ndufs7, Ndufa12, Akt1, Uqcrfs1, Cat, Ndufc2, Pdha1, Ndufs2, Stoml2, Prelid1, Ndufv2, Ndufb10, Ndufv1, Cisd1, Sdhaf4, Pnpt1, Adsl, Slc25a25, Dnajc15, Slc25a33, Etfa, Fxn, Sod2, Nfatc3, Ndufb3, Arl2, Ak4, Noa1, Chchd10			
Oxidative Phosphorylation	Cycs, Ndufa9, Cox5b, Sdhb, Ndufs8, Atp5d, Uqcrc1, Sdha, Ndufa8, Iscu, Cyc1, Gadd45gip1, Ndufab1, Cox6a1, Ndufb6, Ndufb8, Uqcc2, Ndufs5, Sdhc, Ndufs7, Ndufa12, Uqcrfs1, Ndufc2, Ndufs2, Stoml2, Ndufv2, Ndufb10, Ndufv1, Dnajc15, Slc25a33, Fxn, Ndufb3, Ak4, Chchd10	34	34	5.8E-06
Mitochondrial Respiratory Chain Complex Assembly	Ndufa9, Samm50, Ndufs8, Ndufa8, Coa3, Ndufab1, Ndufb6, Ndufb8, Cox16, Uqcc2, Ndufs5, Ndufs7, Ndufa12, Cox17, Ndufc2, Ndufs2, Ndufb10, Sdhaf4, Ndufaf7, Slc25a33, Ndufb3	21	21	4.3E-03
Response to Oxidative Stress	Eif2s1, Ndufs8, Rnf7, Prdx5, Gpx1, Pycr2, Aldh2, Gfer, Rwdd1, Psmb5, Keap1, Diablo, Trap1, Apex1, Tmem161a, Ngfr, Sesn2, Selenon, Mapk13, Endog, Ndufa12, Mapkap1, Akt1, Trp53, Xrcc1, Oxr1, Sesn3, Bag5, Net1, Cat, Plk3, Oxsr1, Ndufs2, Prdx4, Ppp2cb, Pnpt1, Abcc1, Zfp580, Aldh3a2, Sigmar1, Lancl1, Akr1b3, Stk25, Ercc6, Mapk9, Fxn, Hsf1, Ccs, Sod2, Fzd1, Gclc, Axl, Tor1a, Fbxw7, Psip1, Jun, Arl6ip5, Hmox2, Nr4a2	59	59	1.2E-02
