# Supplementary material for: Single-cell analysis of gene regulatory networks in the mammary glands of P4HA1-knockout mice
Source: PLoS Genet. 2025 Jul 22;21(7):e1011505. doi: 10.1371/journal.pgen.1011505 (PMC12310035; doi:10.1371/journal.pgen.1011505)
Supplement: S6 Table — (PDF) [file pgen.1011505.s014.pdf]

S6 Table: Enriched functional groups of genes among DEGs in subcluster U1_wt of the 5Ht mice.

(A) DEGs participating in important (non-inflammatory/immune) biological processes

Enriched GO/KEGG Terms	DEGs	# of DEGs	# of DEGs upregulated in 5Ht	Adjusted p-val
Collagen Metabolic Process	Ctsb, Ctss, Cst3, Vim, Pepd, Tgfb1, Mmp19, Vsir, Il6ra, Rcn3, Mmp2, Ccl2, Adam15	13	12	1.2E-03
Stem Cell Differentiation	Zeb2, Nrp1, Zfp36, Ednrb, Cited2, Vsir, Gsk3b, Rbpj, Lama5, Setd2, Ufl1, Coro1c, Tcof1, Fgfr2, Ap2a2, Ncoa3, Mapk3, Smo	18	16	8.0E-03
Regulation of Cell Morphogenesis	Csf1r, Coro1a, Plxnc1, Actr2, Plxnd1, Picalm, Cyfip1, Gas7, Ccl24, Diaph1, Lst1, Marcks, Ccl7, Arap1, Rhoq, Arhgap18, Coro1c, Mapk9, Eps8, Arhgdia, Shroom3	21	20	3.0E-03
Angiogenesis	Syk, Nrp1, C3ar1, Grn, Cfh, Itgb2, Cybb, Klf2, Plxnd1, Gpx1, C5ar1, Ccl24, Adgrg1, Glul, Stab1, Adam8, Tnf, Mmp19, Ctsh, Erap1, Prcp, Lgals3, Rbpj, Sp1, Setd2, Otulin, Mmp2, Ccl2, Fgfr2, Lemd3, Pik3c2a, B4galt1, Elk3, Adam15	34	31	9.0E-04

(B) DEGs participating in inflammatory and immune responses, as well as macrophage activation and migration.

Enriched GO/KEGG Terms	DEGs	# of DEGs	# of DEGs upregulated in 5Ht	Adjusted p-val
Acute Inflammatory response	Alox5ap, Fcer1g, Fcgr3, Ednrb, Cd163, Adam8, Tnf, Ccr5, Prcp, B4galt1	10	10	7.3E-03
Positive Regulation of Macrophage Migration and Chemotaxis	Csf1r, C3ar1, Ptprj, C5ar1, Ccl2, Mapk3	6	6	4.6E-03
Macrophage Activation	C1qa, Syk, Csf1r, Tyrobp, Ctsc, Grn, Itgam, Tmem106a, Slc11a1, C5ar1, Cd84, Aif1, Ifngr1, Tnf, Tlr2, Pla2g4a	16	16	6.2E-06
B cell Mediated Immunity	Cd74, H2-Aa, H2-Ab1, C1qa, H2-Eb1, C1qb, C1qc, Cfh, Fcer1g, Nfkbiz, Fcgr3, H2-DMb1, Ptpn6, Tnf, Tgfb1, Fgl2, Cd81, Tfrc, Thoc1, H2-DMa, Crlf2	21	21	3.5E-07

T cell Mediated Immunity	Lilrb4a, Slfn2, Ctsc, Unc93b1, Nfkbiz, H2-D1, Slc11a1, Nckap1l, Ctsh, Cd81, H2-K1, Vsir, H2-T23, Crlf2, Hprt	15	15	1.2E-05
