# Supplementary material for: Single-cell analysis of gene regulatory networks in the mammary glands of P4HA1-knockout mice
Source: PLoS Genet. 2025 Jul 22;21(7):e1011505. doi: 10.1371/journal.pgen.1011505 (PMC12310035; doi:10.1371/journal.pgen.1011505)
Supplement: S7 Table — (PDF) [file pgen.1011505.s015.pdf]

S7 Table: Significantly different regulons and the enriched functional groups of genes among their DETGs in subcluster S3 of the 5Ht and 6Ho mice.

(A) Significantly different regulons and their DETGs in subcluster S3 of the 5Ht mice.

TF	(#) DETGs involved in key signaling pathways	Key signaling pathways	# of the DETGs down-regulated in 5Ht	Adjusted p-val
E2f1(+)	(11) Opa3, Sdhaf4, Higd1a, Sesn2, Mrpl15, Tspo, Grpel1, Acaa2, Tomm5, Ndufs8, Opa1	Mitochondrion Organization	11	0.01
	(14) B4galnt1, Tyms, Dut, Tspo, Ndufv2, Tk1, Ran, Dpagt1, Acaa2, Dpy19l1, Nme1, Ndufs8, Opa1, Fasn	Carbohydrate Derivative Metabolic Process	14	0.04

Note: (#) indicates the number of genes involved in key signaling pathways that are enriched among the DETGs.

(B) Significantly different regulons and their DETGs in subcluster S3 of the 6Ho mice.

TF	(#) DETGs involved in key signaling pathways	Key signaling pathways	# of the DETGs up-regulated in 6Ho	Adjusted p-val
E2f1(+)	(11) Dimt1, Utp18, Krr1, Rrp7a, Ddx10, Ercc6, Ftsj3, Trmt112, Lyar, Utp4, Pelo	rRNA Metabolic Process	11	0.04
	(20) Thop1, Hbs1l, Mrps23, Eif4g1, Aplp1, Vars, Mrpl15, Eif3d, Mrpl39, Ifngr1, Becn1, Dph3, Mrrf, Eif2s1, Tsfm, Tyms, Mrpl58, Chchd1, Gclc, Pelo	Peptide Metabolic Process	20	0.08
Zbtb10(+)	(5) Mapkap1, Akt3, Fzd1, Fzd5, Wnt11	mTOR Signaling Pathway	5	0.007
	(5) Ep300, Fzd1, Fzd5, Wnt11, Lzts2	Wnt Signaling Pathway	5	0.008
Rela(+)	(4) Wnt10a, Fzd1, Dvl3, Rheb	mTOR Signaling Pathway	4	0.003
	(3) Wnt10a, Fzd1, Dvl3	Wnt signaling pathway	3	0.014

Note: see legend in (A)
