# Supplementary material for: Single-cell analysis of gene regulatory networks in the mammary glands of P4HA1-knockout mice
Source: PLoS Genet. 2025 Jul 22;21(7):e1011505. doi: 10.1371/journal.pgen.1011505 (PMC12310035; doi:10.1371/journal.pgen.1011505)
Supplement: S8 Table — (PDF) [file pgen.1011505.s016.pdf]

S8 Table: Significantly different regulons and the enriched functional groups of genes involved in collagen metabolic process and stem cell differentiation among their DETGs in subcluster U1_wt of the 5Ht mice.

(A) Collagen metabolic and catabolic processes

TF	(#) DETGs involved in collagen metabolic and catabolic processes	# of the up-regulated DETGs	Adjusted p-val
Spi1 (+)	(3) Ctss, Cst3, Mmp19	3	0.04
Mitf(+)	(3) Il6ra, Ctss, Vsir	3	0.07

Note: (#) indicates the number of genes involved in collagen metabolic and catabolic processes that are enriched among DETGs in subcluster U1_wt of the 5Ht mice.

(B) Stem cell differentiation

TF	(#) DETGs involved in stem cell development and differentiation	# of the up-regulated DETGs	Adjusted p-val
Mitf (+)	(5) Zeb2, Ednrb, Nrp1, Rbpj, and Vsir	5	0.03

Note: (#) indicates the number of genes involved in stem cell development and differentiation that are enriched among DETGs in subcluster U1_wt of the 5Ht mice.
