# Supplementary material for: Single-cell analysis of gene regulatory networks in the mammary glands of P4HA1-knockout mice
Source: PLoS Genet. 2025 Jul 22;21(7):e1011505. doi: 10.1371/journal.pgen.1011505 (PMC12310035; doi:10.1371/journal.pgen.1011505)
Supplement: S9 Table — (PDF) [file pgen.1011505.s017.pdf]

S9 Table: Significantly different regulons and the enriched functional groups of genes among their DETGs in subcluster U1_wt of the 5Ht mice.

TF	DETGs involved in inflammatory responses			DETGs involved in macrophage activation and chemotaxis		
	(#) DETGs	# of the up-regulated DETGs	Adjusted p-val	(#) DETGs	# of the up-regulated DETGs	Adjusted p-val
Fli1(+)	(32) Sgms1, Fcer1g, C5ar1, Pld4, Slc11a1, Ccl24, Stab1, Rbpj, Ctsc, C1qa, Adam8, Ednrb, Nrros, Ccl7, Alox5ap, Ccl9, Nfkbid, Itgam, Cxcl2, Prcp, Clec10a, Pf4, Syk, Cd163, Ly86, Ccl6, Cybb, Csf1r, Ptafr, Sirpa, Adam17, Gpx1	32	1.2E-11	(7) C5ar1, Slc11a1, Ctsc, C1qa, Itgam, Syk, Csf1r	7	2.4E-03
Spi1(+)	(47) Pf4, Alox5ap, Ednrb, Fcer1g, Nfkbid, Ctss, Syk, Ccl24, Gpx1, Cxcl2, Ccl4, Itgb2, Fcgr3, Nfkbiz, Igf1, Slc11a1, Tyrobp, Cybb, Clec10a, Ccl6, Ccl9, Cd14, Cd163, Tlr2, Pld4, Ctsc, Csf1r, C1qa, C5ar1, Zfp36, Cyba, Lilrb4a, Lpl, Ly86, Cfh, Stab1, Ptafr, Ifngr1, Itgam, Tnfaip3, Adam8, Rbpj, Aif1, C3ar1, Lyn, B4galt1, Nrros	47	1.7E-18	(12) Syk, Slc11a1, Tyrobp, Tmem106a, Tlr2, Ctsc, Csf1r, C1qa, C5ar1, Ifngr1, Itgam, Aif1	12	1.0E-06
Mitf(+)	(14) Ednrb, Stab1, C1qa, Itgb2, Cfh, Cybb, Ctss, Lilrb4a, Ccl6, Syk, Rbpj, Csf1r, Ctsc, Rel	14	1.6E-05	(4) C1qa, Syk, Csf1r, Ctsc	4	2.0E-02
Irf8(+)	(33) Pf4, Nfkbid, Fcgr3, Tlr2, Clec10a, Lpl, Fcer1g, Ccr5, C1qa, Ccl2, Ctss, Ccl4, Syk, Ccrl2, Ccl24, Cfh, Alox5ap, Ptafr, Ly86, Tyrobp, Aif1, C3ar1, Cd163, Cd14, Cxcl2, Rel, Ccl9, Cybb, Ednrb, C5ar1, Itgam, Ifngr1, Slc11a1	33	1.9E-12	(10) Tlr2, C1qa, Syk, Tyrobp, Aif1, Cd84, C5ar1, Itgam, Ifngr1, Slc11a1	10	5.6E-06
Irf4(+)	(17) Lpl, Tnfaip3, Fcgr3, C1qa, Ccr5, Tnf, Prcp, Ptafr, Nrros, Ccl4, Nfkbid, Ccrl2, Ctsc, Ccl7, Ly86, C3ar1, Nfkbiz	17	8.6E-04			
Irf5(+)	(23) Tnfaip3, Itgb2, Cxcl2, C1qa, Nfkbid, Ccl6, Ednrb, Ctss, Rbpj, Lpl, Ccl7, Cyba, C5ar1, Ccl9, Ccrl2, Slc11a1, Ly86, Cybb, Alox5ap, Pld4, Fcer1g, Adam8, Cd163	23	8.5E-09			

Irf7(+)	(17) Cd14, Tnfaip3, Fcer1g, Lilrb4a, Syk, Itgam, Itgb2, Tyrobp, Alox5ap, Sgms1, Sirpa, C3ar1, Tlr2, Nfkbiz, Rel, Lyn, Pf4	17	3.6E-05	(4) Syk, Itgam, Tyrobp, Tlr2	4	4.0E-02
Stat1(+)	(14) Sirpa, Nfkbid, Pf4, Ednrb, Alox5ap, Cfh, Slc11a1, Ccl6, Tnfaip3, Itgam, Syk, Adam8, Lpl, Ccl9	14	9.5E-04	(3) Ednrb, Lgals3, Ptprj	3	2.7E-02
Rel(+)	(22) Nfkbiz, Lyn, Nfkbia, Ednrb, Ccl7, Fcer1g, Nrros, Ctss, Tnfaip3, Pld4, Cd14, Syk, Tlr2, Adam8, Cfh, Cd163, Tnf, Itgam, Stab1, C1qa, Ifngr1	22	3.8E-06	(7) Syk, Tlr2, Tmem106a, Tnf, Itgam, C1qa, Ifngr1	7	8.7E-04
Klf2(+)	(19) Ctsc, Tnfaip3, C1qa, Ccl24, Lpl, Clec10a, Cd163, Sgms1, Nfkbiz, Ednrb, Zfp36, Ptafr, C5ar1, Nrros, Ccr5, Slc11a1, Pf4, Nfkbia, Ccl9	19	1.1E-05	(4) Ctsc, C1qa, C5ar1, Slc11a1	4	7.8E-02
Jund(+)	(18) Nfkbiz, Ccl4, Cxcl2, Ccl7, Adam8, Stab1, C5ar1, Ccrl2, Csf1r, Cd14, Fcer1g, Itgb2, Nrros, C1qa, Ccl9, Tnf, Lilrb4a, Cfh	18	8.7E-06	(5) Cd84, C5ar1, Csf1r, C1qa, Tnf	5	2.9E-02
Runx3(+)	(6) Lpl, Lyn, Nfkbiz, Gpx1, Ctss, Slc11a1	6	2.2E-02			
Maf(+)	(19) Sgms1, Ccl7, Ctsc, Nfkbid, C5ar1, Syk, Lpl, Fcgr3, Ly86, Igf1, Cd14, C3ar1, Pld4, Alox5ap, Fcer1g, Ccl9, Prcp, Cxcl2, Nfkbiz	19	4.2E-04			
Pparg(+)	(4) Rac2, Ptpn6, Dock2, Tyrobp	4	5.6E-02			
Stat2(+)	(14) Setd2, Hexim1, Cyba, Cd36, Tyrobp, Ccl7, Syk, Ccl6, C1qa, C1qb, Bst2, Unc93b1, Mndal, Arhgef2	14	7.8E-05			
